# Omics approaches revealed how arbuscular mycorrhizal symbiosis enhances yield and resistance to leaf pathogen in wheat

**DOI:** 10.1038/s41598-018-27622-8

**Published:** 2018-06-25

**Authors:** Valentina Fiorilli, Candida Vannini, Francesca Ortolani, Daniel Garcia-Seco, Marco Chiapello, Mara Novero, Guido Domingo, Valeria Terzi, Caterina Morcia, Paolo Bagnaresi, Lionel Moulin, Marcella Bracale, Paola Bonfante

**Affiliations:** 1 Department of Life Sciences and Systems Biology, Università degli Studi di Torino, Viale P.A. Mattioli 25, 10125 Torino, Italy; 2 Dipartimento di Biotecnologie e Scienze della Vita, Università degli Studi dell’Insubria, via J.H. Dunant 3, 21100 Varese, Italy; 3 IRD, Cirad, Univ. Montpellier, Interactions Plantes Microorganismes Environnement (IPME), 34394 Montpellier, France; 4 CREA-GB, Research Centre for Genomics and Bioinformatics, Via San Protaso 302, 29017 Fiorenzuola d’Arda, Italy

## Abstract

Besides improved mineral nutrition, plants colonised by arbuscular mycorrhizal (AM) fungi often display increased biomass and higher tolerance to biotic and abiotic stresses. Notwithstanding the global importance of wheat as an agricultural crop, its response to AM symbiosis has been poorly investigated. We focused on the role of an AM fungus on mineral nutrition of wheat, and on its potential protective effect against *Xanthomonas translucens*. To address these issues, phenotypical, molecular and metabolomic approaches were combined. Morphological observations highlighted that AM wheat plants displayed an increased biomass and grain yield, as well as a reduction in lesion area following pathogen infection. To elucidate the molecular mechanisms underlying the mycorrhizal phenotype, we investigated changes of transcripts and proteins in roots and leaves during the double (wheat-AM fungus) and tripartite (wheat-AM fungus-pathogen) interaction. Transcriptomic and proteomic profiling identified the main pathways involved in enhancing plant biomass, mineral nutrition and in promoting the bio-protective effect against the leaf pathogen. Mineral and amino acid contents in roots, leaves and seeds, and protein oxidation profiles in leaves, supported the *omics* data, providing new insight into the mechanisms exerted by AM symbiosis to confer stronger productivity and enhanced resistance to *X*. *translucens* in wheat.

## Introduction

In natural environments, plants interact simultaneously with a broad spectrum of both pathogenic and beneficial microorganisms that might influence plant performance and survival. Among soil microbes, arbuscular mycorrhizal (AM) fungi (subphylum Glomeromycotina^1^) establish a symbiosis with most plants living in wild and agroecosystems^2^. AM fungi colonise the root cortex, supplying mineral nutrients to plants in exchange for carbon compounds, thanks to the development of highly branched intracellular structures called arbuscules^3^. Besides improved mineral nutrition, plants colonised by AM fungi often display increased biomass and yield grain and a higher tolerance to biotic and abiotic stresses leading to a general improvement in plant fitness^4–7^. Considering the range of benefits provided by the fungal partner, the management of AM fungi in crop production is a cornerstone for future low-input and sustainable agriculture.

Many studies have focused on local and systemic transcriptomic and proteomic changes in rice^8–10^, maize^11^, *Medicago truncatula*^12,13^) and tomato plants^14–16^. By contrast, even though wheat (*Triticum aestivum* L.) is a major global crop, cultivated on more than 200 million hectares with more than 700 million tons of annual production^17^, its response to AM symbiosis has been poorly investigated. The main reason for this backwardness is that wheat has a hexaploid genome of 17 Gb in size, more than 80% of which is composed of repetitive transposable elements^18^. It is considered one of the most challenging genomes, since it has the genetic structure of three independent genomes in one species (*AABBDD* genome)^19^. A meta-analysis highlights the beneficial effects of mycorrhizal inoculation on wheat dry weight and phosphorus (P), nitrogen (N) and zinc (Zn) uptake^20^. However, the molecular determinants underlying the AM-related growth promotion and enhanced nutrient status in wheat are still poorly understood.

AM symbiosis is acknowledged to reduce damage caused by soil-borne pathogens including fungi, oomycetes^21^ and parasitic nematodes^22,23^. Similarly, in wheat, where pathogen attacks cause about 10–16% yield losses^24^, some of these stress events are alleviated by AM fungi^25^. The mechanisms involved in the bio-protective effect of AM fungi are not fully explained: they are not exclusively dependent on the improved mineral nutrition, but seem to be related to activation of plant defence mechanisms^26^. In addition, plant hormones and small RNA molecules (sRNAs) are attractive candidates for long-distance defence signals^6^. Plant hormones and small RNA molecules (sRNAs) are attractive candidates for long-distance defence signals^6^. Indeed, establishment of an AM symbiosis and production of AM signals activate defence-responsive genes in both shoot and root^12,27–29^. This boost of basal defences is known as priming, and it could be successfully triggered by various natural and artificial compounds, including AM fungi^30^. As a consequence, mycorrhizal plants are expected to be better protected against pathogen challenge than non-mycorrhizal plants: this phenomenon has been named *mycorrhiza-induced resistance* (MIR)^4,6^. MIR is dependent on the particular pathogen-mycorrhizal plant interaction and the plant organ under examination, i.e., root or shoot^12,21,31–34^.

The contrasting results obtained in such studies suggest that induction of resistance against pathogens depends on multiple mechanisms that may operate simultaneously^4,31,33,34^. The potential protective effect of mycorrhizal symbiosis in wheat has been poorly investigated^35,36^. The main goals of this work were, first, to define the responsiveness of *T*. *aestivum* cv. Chinese Spring to AM symbiosis, and second, to elucidate the molecular mechanisms underlying the mycorrhizal phenotype. We looked for the main pathways involved in enhancing plant biomass and mineral nutrition, and in promoting the bio-protective effect against a leaf pathogen. To address these issues, we combined phenotypic, and molecular metabolomic approaches. We explored the plant growth effect exerted by AM fungi in both greenhouse and controlled environment conditions, and further evaluated the impact of AM symbiosis against *Xanthomonas translucens*, which is a specific pathogen of wheat leaves. By integrating whole-transcriptome sequencing (RNA-seq) with shotgun nanoflow scale liquid chromatography-tandem mass spectrometry (LC-MS/MS), we provide a comprehensive functional overview of both local and systemic transcriptomic and proteomic changes in roots and leaves during the mycorrhizal combination (plant and AM fungus), as well as during the tripartite interaction (plant, AM fungus and pathogen). Mineral and amino acid contents in roots, leaves and seeds, and protein oxidation profiles in leaves, supported the *omics* data, providing new insight into the mechanisms exerted by AM symbiosis to confer positive effects on wheat development, and resistance to a wheat pathogen.

## Results

### Greenhouse experiment

Table 1 presents the results of a 2 year greenhouse trial to evaluate the impact of *Funneliformis mosseae* inoculation in Chinese Spring wheat. ANOVA analysis showed that all measured agronomic traits, such as tillering capacity, vegetative biomass and yield, as well as qualitative traits, such as kernel weight and size, are significantly different between mycorrhizal (M) and non-mycorrhizal control (C) plants. The presence of *F*. *mosseae* correlated with greater tillering capacity and plant biomass and also with increased yield, kernel weight and size (Fig. S1a). AM fungal inoculation also led to significant increases in concentrations of P, Mg and Zn in M seeds. In addition, total amino acid (AA) content increased (methionine, ornithine, tyrosine and tryptophan were more abundant), while lysine content decreased (Fig. S1b,c).

**Table 1:** The mean values of agronomic and qualitative traits are reported.

Agronomic traits	Treatments	
	Control	Myc
Number of tillers/plant	9.95 ± 2.5	11.25 ± 1.86
Vegetative tissue DW (g pot^−1^)	12.9 ± 5	17.6 ± 5.1
Grain yield (g pot^−1^)	5.5 ± 2.4	8.4 ± 2.4
Thousand kernel weight (g)	30.15 ± 3.8	35.7 ± 2.4
Kernel area (mm^2^)	15.06 ± 1.01	16 ± 0.37
Kernel major ellipse (mm)	5.7 ± 0.2	5.8 ± 0.1
Kernel minor ellipse (mm)	2.7 ± 0.2	2.9 ± 0.1
*F*. *mosseae* detection in root tissue by qRT-PCR	−	+

Sixty control and sixty *F*. *mosseae* inoculated plants were grown in greenhouse. The mycorrhization of root tissues have been evaluated with qPCR assay and the results obtained are reported as absence (−) or presence (+) of *Funneliformis mosseae* DNA. ANOVA analysis showed that all the measured agronomic traits, are significantly different between control and mycorrhizal (myc) plants.

### Phenotypic assessment under controlled conditions

**Figure 1:**
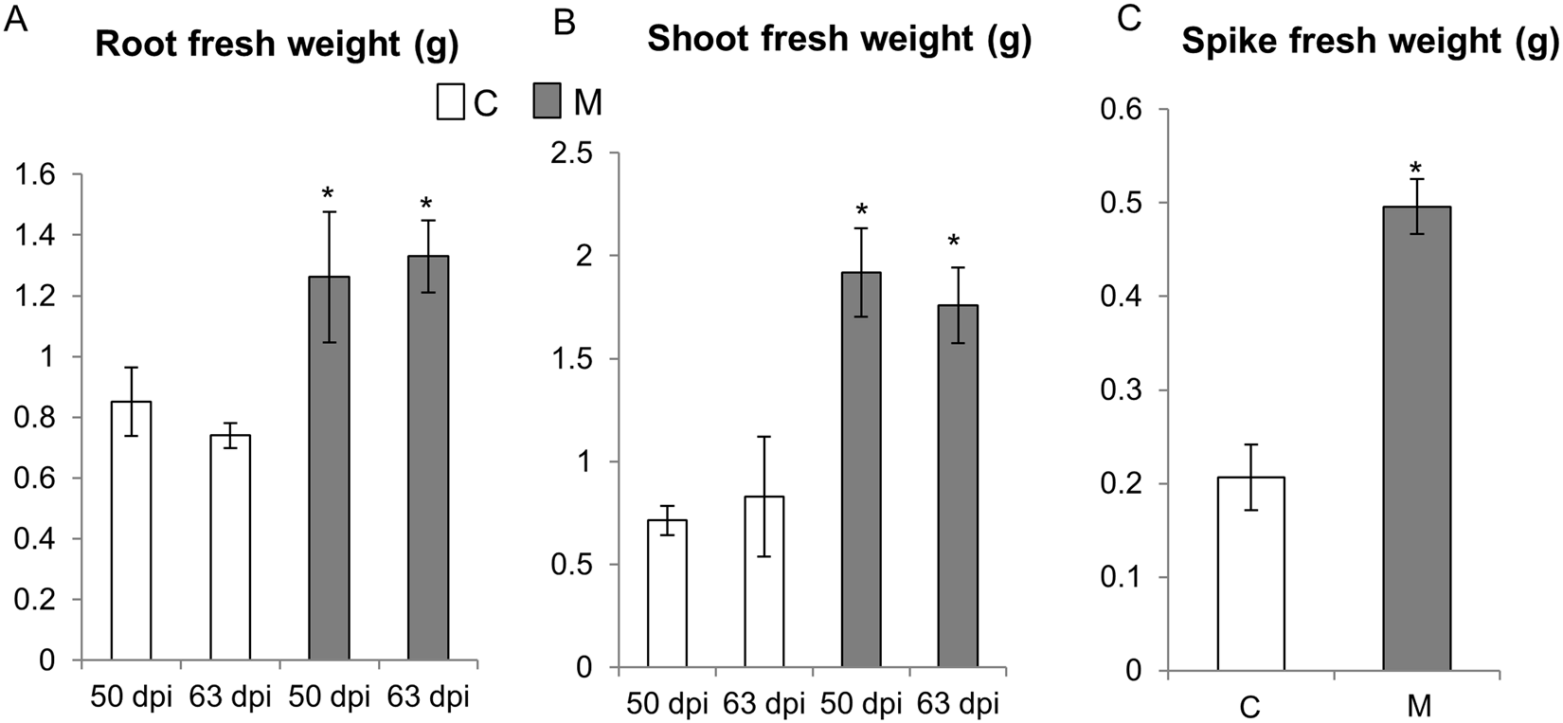
Effect of AM symbiosis on wheat biomass in different plant organs. Fresh weight of roots (**A**) and leaves (**B**) of the control (C) and mycorrhizal (M) wheat plants harvested at 50 and 63 days post AM fungus inoculation. (**C**) Spike fresh weight of control and mycorrhizal plants evaluated at the end of wheat natural life cycle. Data (means ± SD, n ≥ 6) were subjected to one-way analysis of variance (ANOVA). The asterisks indicated significant differences at the 5% level using Tukey’s test.

To confirm the morphometric data recorded in the greenhouse, C and M plants were grown in controlled conditions in a growth chamber, and the biomass of their epigeous and hypogeous parts was measured at 50 and 63 dpi. Growth of both tissues was increased significantly in M plants compared with C plants (Fig. 1A, B). To better investigate the effect of AM symbiosis on plant yield, spike weight was evaluated in M and C plants at the end of their natural life cycle. M plants displayed higher spike weight than C plants (Fig. 1C).

**Figure 2:**
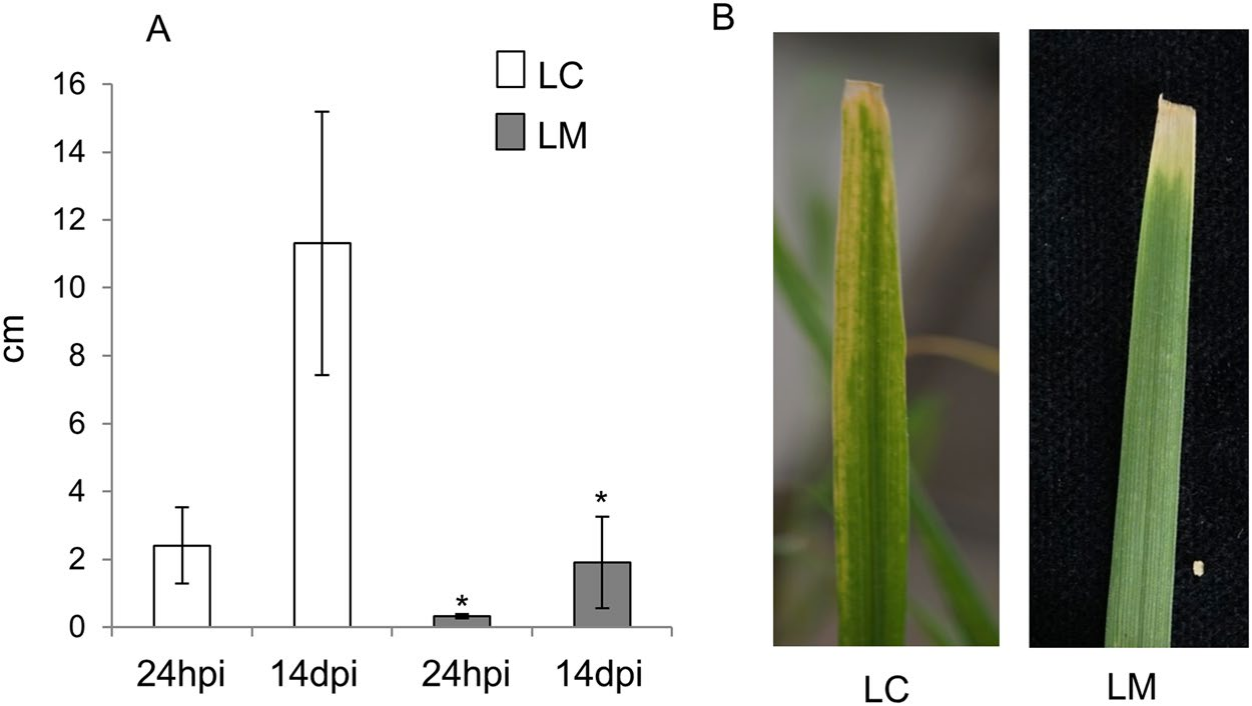
Phenotypic evaluation of disease symptoms caused by the bacterial pathogen *Xanthomonas translucens* in control (C) and mycorrhizal (M) plants. (**A**) Disease area (cm) was assessed on leaves from control (LC) and mycorrhizal (LM) plants 24 h post inoculation (hpi) and 14 days post inoculation (dpi). (**B**) The pictures show lesions provoked by *X*. *translucens* on LC and LM 14 dpi. Data (means ± SD, n ≥ 6) were subjected to one-way analysis of variance (ANOVA). The asterisks indicated significant differences at the 5% level using Tukey’s test.

In the same experiment, 12 pots were devoted to investigating the impact of the AM fungus on leaf infection with *X*. *translucens*. These plants were identified as MX, while control plants infected with the pathogen were identified as CX. For all samples, mycorrhizal success was evaluated 50 days post inoculation (dpi) by calculating the total length of root colonisation (F%) and the total number of arbuscules (A%) in M plants (F%: 53.5 ± 16.9; A%: 26.3 ± 9) and in MX plants (F%: 59.7 ± 12.6; A%: 34.5 ± 14.2). Similar colonisation values were detected 63 dpi in M (F%: 61.5 ± 16.3; A%: 38.5 ± 10.2) and MX plants (F%: 60.5 ± 19.2; A%: 40.5 ± 12.2), revealing that pathogen inoculation of the leaves did not inhibit root colonisation by the AM fungus, in the short term. At the same time (63 dpi, i.e., 14 days after inoculation with *X*. *translucens*), disease symptoms were evident. Lesion length was significantly reduced in MX plants compared with CX plants (Fig. 2A,B).

These experiments demonstrate that AM symbiosis exerts a positive effect on wheat growth and provides protection against *X*. *translucens*.

### A quantitative overview of transcript and proteomic data sets

RNAs and proteins were isolated from leaves (L) and roots (R) of wheat plants, grown in the absence (LC and RC) or in the presence of the mycorrhizal fungus *F*. *mosseae* (LM and RM), and following infection with the bacterial pathogen *X*. *translucens* (LMX and RMX).

For transcriptomic analysis, each treatment was sequenced in triplicates, with 37 million reads on average per replicate, and a minimum of 27 million and maximum of 41 million reads per replicate. Pearson correlation coefficients for biological replicate samples sharing the same treatment and tissue were always above 0.9 (Table S3). Also, for proteomic investigation, each treatment was analysed in triplicate leading to 2,750 proteins identified on average per replicate, with a minimum of 2,659 and a maximum of 2,800 proteins per replicate. Pearson correlation coefficients for biological replicate samples sharing the same treatment and tissue ranged from 0.95 to 0.99 (Table S4). All genes with a false discovery rate (FDR) below 0.05 and log_2_FC over 0.5 and proteins with FDR below 0.01 and log_2_FC over 0.3 were considered differentially expressed (differentially expressed genes, DEGs; and differentially expressed proteins, DEPs).

**Figure 3:**
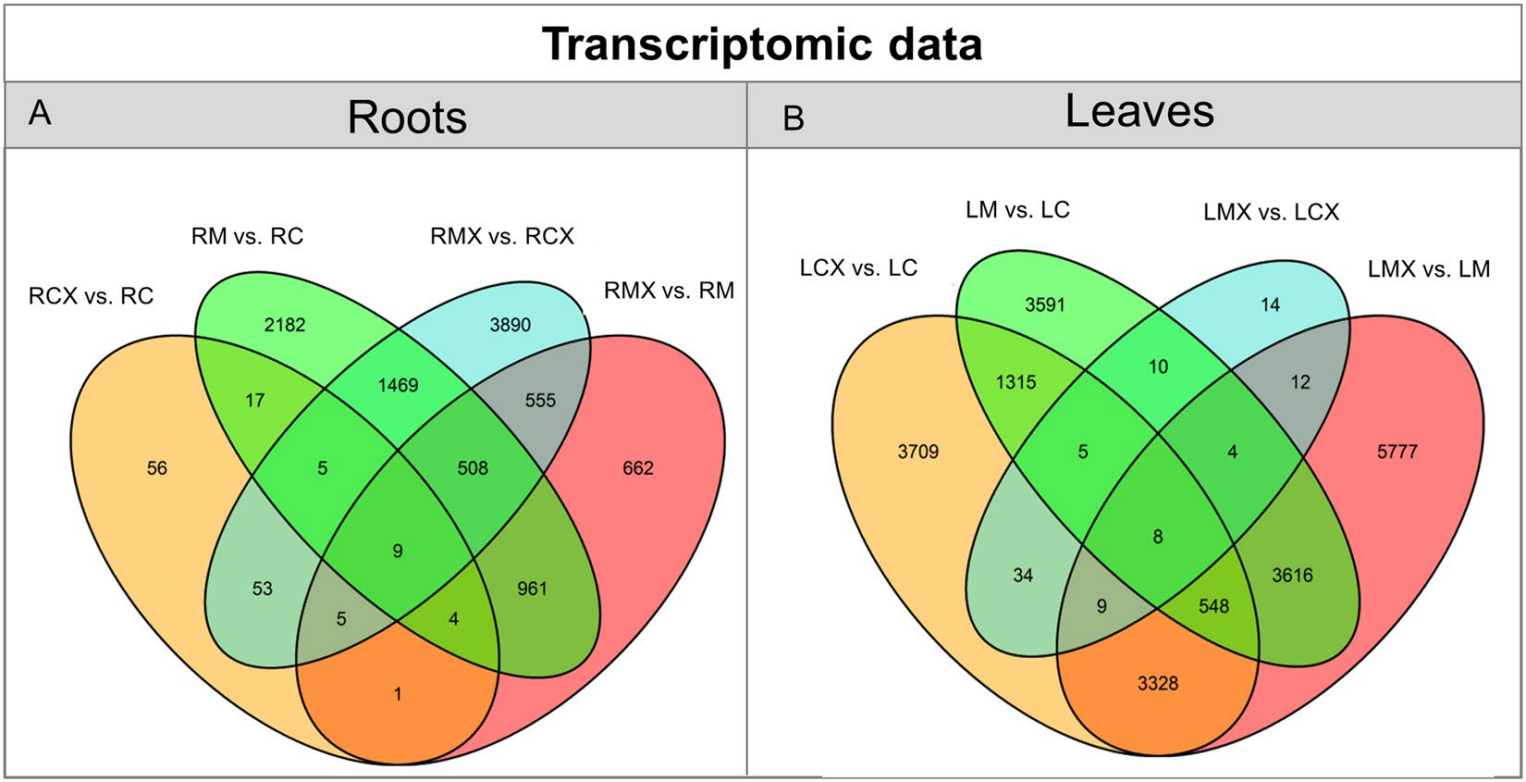
Venn diagrams of DEGs modulated in the different comparisons in roots and leaves. Venn diagrams illustrating the relationships between DEGs in the different contrasts among the same organ (**A**) roots and (**B**) leaves in the absence (C) or presence (M) of the AM fungus and following (CX; MX) or not pathogen infection.

The overall changes in gene expression detected in the different comparisons are represented in a Venn diagram (Fig. 3). As expected, the AM fungus had a greater impact on the root system than bacterial infection (RM *vs* RC: 5,155 DEGs and RCX *vs* RC: 150 DEGs, respectively). The AM fungus had a deep impact on the leaf profile (LM *vs* LC: 9,097 DEGs) as well as the bacterial inoculation (LCX *vs* LC: 8,408 DEGs). The presence of *Xanthomonas* on leaves of mycorrhizal plants (LMX *vs* LM) led to a higher number of DEGs (13,302) than other comparisons, and 43% (5,777) of these DEGs were exclusively regulated in this contrast (Fig. 3B), suggesting that a synergistic effect occurs. As a consequence of the huge number of DEGs in LMX and LCX, their direct comparison led to a low DEGs number (97).

Overall, these data reveal that the AM fungus has a strong local and systemic impact, while the pathogen exerts a local effect during the binary interaction, and both local and systemic ones when inoculated on mycorrhizal plants.

**Figure 4:**
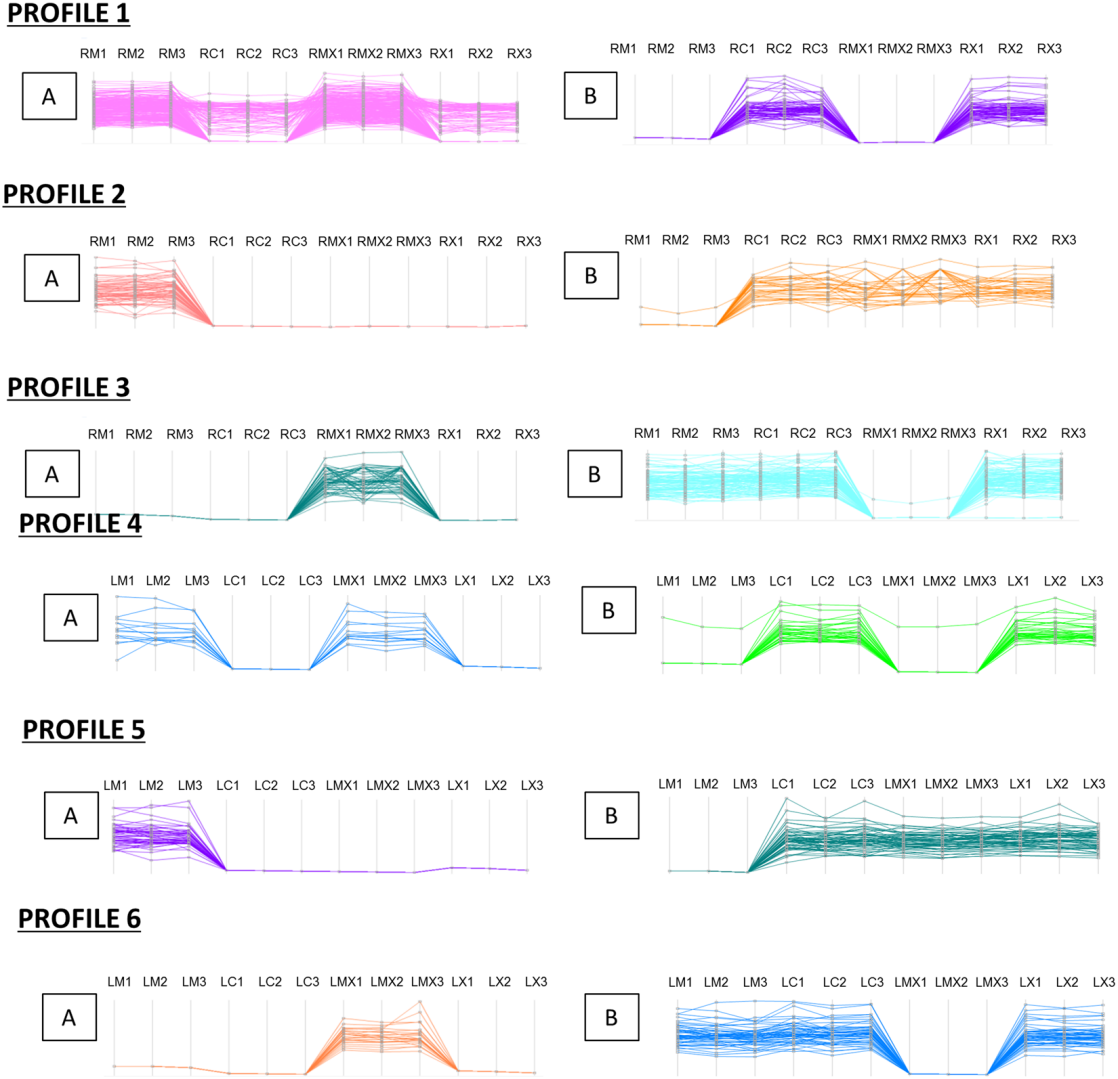
Protein profiles of roots and leaves. For each sample three biological replicas were considered (indicated as 1, 2, 3). (**A** and **B**) Indicate profiles containing respectively up or down regulated proteins in mycorrhizal samples (RM, RMX, LM, LMX).

For a deeper analysis, we compared the expression of all identified proteins in the four samples (M, C, MX, X) by cluster analysis, on the basis of the modulation of their expression. Figure 4 shows some of the protein profiles obtained.

### Differentially regulated genes and proteins in wheat leaves and roots following colonisation by *F*. *mosseae*

**Figure 5:**
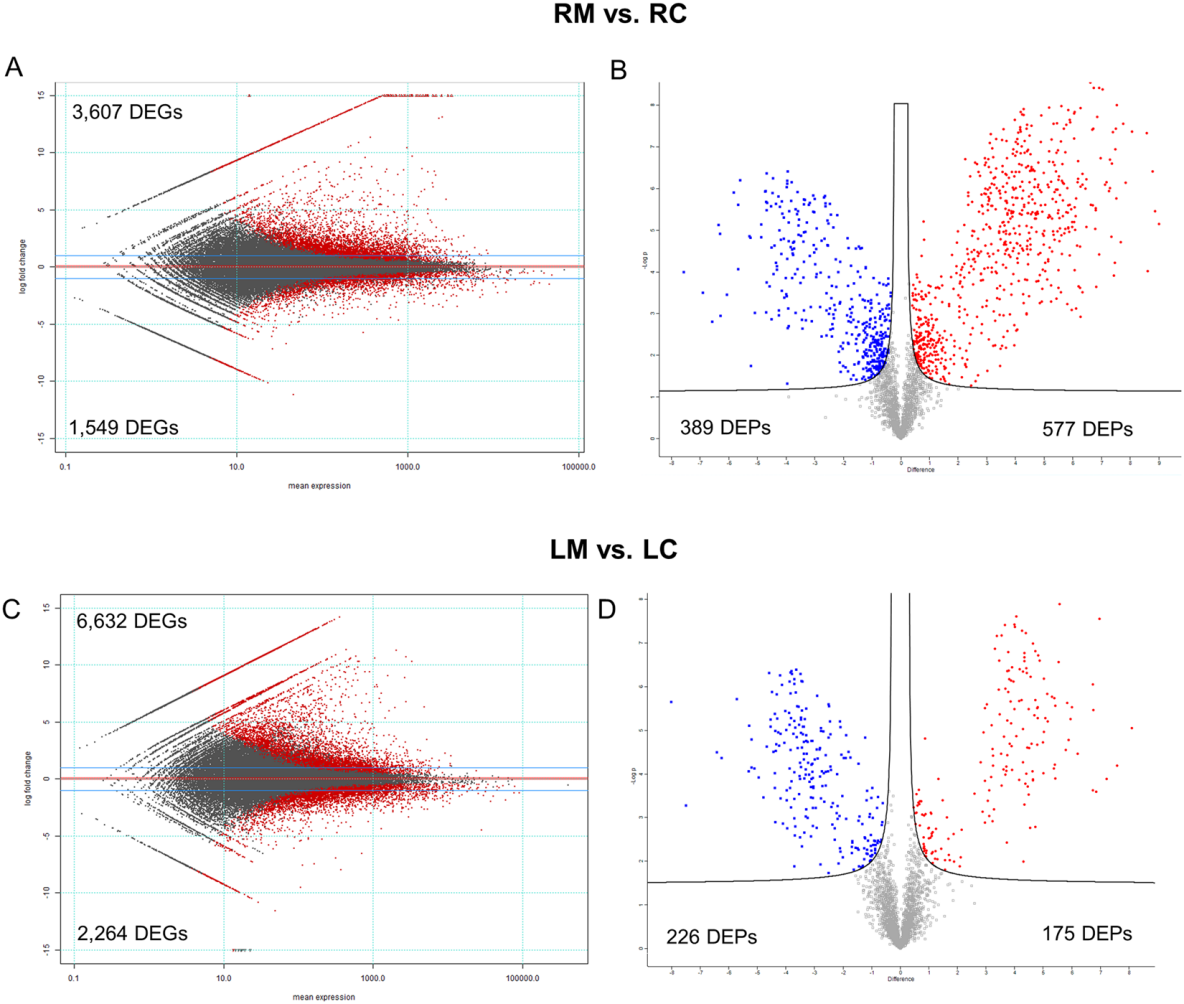
Global overview of the transcriptional and proteomic changes in the two organs (leaves -L and roots- R) in the absence (C) or presence (M) of the AM fungus. Mean expression versus log2 fold change plots (MA-plots, left side; Volcano plot, right side) were computed for these comparisons: (**A**,**B**) RM *vs* RC, and (**C**,**D**) LM *vs* LC. Called DEGs (**A**,**C**) and DEPs (**B**,**D**) (FDR 0.05) are plotted in color.

RNA-seq analysis revealed 3,607 up-regulated and 1,549 down-regulated genes in M roots (Fig. 5A and Table S5). An even higher gene modulation was observed in leaves: 6,632 were up-regulated and 2,464 were down-regulated genes (Fig. 5C and Table S6). RNA-seq analysis revealed 532 and 2,220 transcripts that were exclusively expressed in roots (RM *vs* RC) and leaves (LM *vs* LC) of M plants, respectively.

Unlike wheat, rice has been much studied as a model plant for AM symbiosis^9,10,37^. To determine whether a common core of genes responds to AM symbiosis, we compared the available AM-rice root RNA-seq data set^10^ with the wheat data in the present work. Using a Reciprocal Best Hits (RBH), we identified 114 of the 1,088 up-regulated rice genes, which contained a corresponding sequence in mycorrhizal wheat roots (Table S7). Among them were several AM marker genes identified in other AM host plants: Glycerol-3-phosphate acyltransferase (*OsRAM2* homolog)^38^, *Gibberellin response modulator protein* (LjRAD1 homolog)^39^, LysM domain-containing protein (*OsLysM* homolog)^10^, *Ammonium transporter* and *Inorganic phosphate transporters* (see nutrient uptake paragraph) and *ABC-2 type transporter* (*OsSTR1* homolog)^40^ (Table S7). These genomic commonalities further support a key role for these genes in establishment of the AM symbiosis.

When RM samples were compared with RC samples, proteomic analysis revealed 586 up-regulated and 395 down-regulated proteins (Fig. 5B and Table S8), and a comparison between LM and LC samples revealed 175 up-regulated and 226 down-regulated proteins (Fig. 5D and Table S9).

To gain an overview of the overlap between transcriptomic and proteomic data sets, differentially regulated genes and proteins detected using the two high-throughput techniques were compared. In roots and leaves of mycorrhizal plants, transcriptomic and proteomic data sets shared 192 (3.7% of DEGs and 19% of DEPs) and 82 (0.9% of DEGs and 20% of DEPs) elements, respectively (Fig. S2; Tables S10 and S11).

Taking advantage of bioinformatics tools such as agriGO v2.0^41^ and over-represented Gene Ontology (GO) categories, we identified the pathways elicited by the AM fungus locally (root) and systemically (leaf). In the following paragraphs, we illustrate those pathways that might better explain the effects on growth and bio-protection detected in mycorrhizal plants.

#### Nutrient uptake

Nutrient uptake is a crucial trait of the AM symbiosis; however, in wheat, AM-induced nutrient transporter genes are still poorly characterised. To help plug this knowledge gap, the transcription profiles of some phosphate transporters (PTs), ammonium transporter 3 member 1 (*TaAMT3*.*1*), high-affinity sulfate transporter 2 (*TaSulfTr2*), potassium channel (*TaAKT1*) and oligopeptide transport (*TaOPT*) were investigated by both RNA-seq and qRT-PCR analyses. We monitored transcript levels of the PT genes previously described in wheat as highly induced in M roots (*TaPT10*, *TaPT11* and *TaPT12*)^42,43^, and clustered with the AM-induced PT genes (*OsPT11*; *MtPT4*; *LjPT4*^44–46^; (Fig. S3) as well as the putative inorganic phosphate transporter 1–13 (*TaPT13*), which shows high homology with the AM-induced *OsPT13*^47^ (Fig. S3). All of these PT genes were strongly induced in RM *versus* RC (Fig. S4). The transcripts of *TaAMT3*.*1*, which shows high similarity to the AM-induced *OsAMT3*.*1* from rice^10,48^, were detected exclusively in M roots (Fig. S4). The same gene expression profile was observed for *TaAKT1* and *TaOPT*, whose transcripts were detected only in M roots. By contrast, although *TaSulfTr2* was strongly induced in M roots, it was also expressed in C roots (Fig. S4). A comparable expression profile was detected for *LjSultr1;2* which was induced in *L*. *japonicus* by both sulphur starvation and mycorrhizal formation^49^.

AM colonisation led to the differential regulation of several proteins involved in nutrient uptake. In agreement with the transcriptomic data, TaPT10 and TaAMT3.1 proteins were accumulated in RM. In addition, two proteins involved in N uptake were up-regulated in RM *vs* RC: an AMT which shows high similarity to OsAMT3.2 and a nitrate transporter with similarity to the tomato AM-inducible LeNRT*2*;*3*^50^. Accordingly, two glutamine synthases, involved in N assimilation, were more strongly expressed in RM than in RC.

Several proteins involved in iron (Fe) uptake also accumulated in RM: one Fe-phytosiderophore transporter, some nicotianamine synthases (NAS) and two deoxymugineic acid synthases. Finally, a H^+^-ATPase and a copper (Cu)-transporting ATPase were also induced in RM. H^+^-ATPase shows high homology to OsHa1 and MtHA1, which energise nutrient uptake during mycorrhizal symbioses in rice and *Medicago truncatula*^51^. The Cu-transporting ATPase is 90% similar to HMA4 of *Oryza sativa*, which is involved in Cu accumulation in root vacuoles^52^. To validate the omics data, the mineral profiles of C and M plants were determined in both roots and leaves (Table 2). In agreement with the increased expression of phosphate and Cu transporters, P and Cu contents were significantly higher in RM. Mg, potassium (K) and Fe concentrations were lower in RM and higher in LM suggesting that AM promotes mineral translocation towards the shoot. These results may partially explain the increased expression of NAS in RM, since NAS expression is differentially regulated by Fe status^53^.

**Table 2:** Changes in mineral content in roots and leaves of control and mycorrhizal plants.

Mineral	Unit	Roots	Leaves
		RM/RC	LM/LC
Ca	µg/mg	2.09	**0**.58*
K	µg/mg	**0**.87*	**1**.19*
Zn	µg/mg	0.78	1.19
Cu	ng/mg	**2**.58*	**1**.86*
Mg	µg/mg	**0**.49*	**1**.38*
Fe	µg/mg	0.60	**1**.64*
P	µg/mg	**1**.67*	0.97

Values represent the mineral nutrient content ratio between roots and leaves of control (C) and mycorrhizal (M) plants collected 50 days post AM fungus inoculation (two-tailed *t* test; **P < 0.01 and *P < 0.05).

In conclusion, transcriptomic and proteomic data sets revealed a strong activation of nutrient transporter genes and proteins in M wheat plants, leading to an increased mineral content in both roots and leaves.

#### Primary metabolism and AA content

Both transcriptomic and proteomic data sets revealed differential expression of genes and proteins involved in primary metabolism in roots and leaves of M plants (Figs S5 and S6).

Several genes and proteins involved in carbohydrate metabolism (glycolysis, tricarboxylic acid [TCA], OPP pathways) were induced in RM, to provide energy and carbon skeleton during AM colonisation (Fig. S6). Due to the AM requirement for reduced plant carbon, metabolic pathways producing glucose were expected to be up-regulated^54^. Enzymes involved in sucrose cleaving, such as sucrose synthases, invertases and fructokinases 1 and 2, were indeed up-regulated in wheat M roots. Recent work also demonstrated lipid movement from host plants to AM fungi, a process requiring strong activation of lipid metabolism in the arbuscule-containing cells of mycorrhizal roots in *Medicago*, lotus and carrot^55–58^. Fatty acid biosynthesis was also strongly up-regulated in wheat RM: we detected increased transcripts and protein levels of key enzymes of this pathway, including 3-ketoacyl-CoA synthases, plastid acetyl-CoA carboxylase and 3-oxoacyl-[acyl-carrier-protein] reductase 3. Several other genes and proteins involved in lipid metabolism, including Triacylglycerol lipase 1 precursor, GDSL-like lipase/acylhydrolase, plastid omega-3 fatty acid desaturase, Aestivum stearoyl-ACP desaturase and lipid-transfer protein (LTP35), were strongly induced in RM *vs* RC. At the proteomic level, the accumulation of Acyl-CoA dehydrogenase family member 10, and Glyoxysomal fatty acid beta-oxidation multifunctional protein MFP-a, which are involved in fatty acid beta-oxidation, provided further support for the role of lipid metabolism in the AM symbiosis.

Focusing on the leaves of M plants, and on the list of common DEGs and DEPs, we identified the up-regulation of genes and proteins involved in photosynthesis and related processes. We identified genes and proteins related to RuBisCO large subunit-binding protein, Photosystem II 10 kDa polypeptide, two Sucrose synthases 1 and one cell wall invertase, the last responsible for mediating sucrose cleavage yielding UDP-glucose and fructose. Proteomic results also showed the accumulation of ferrochelatase 2 (FC2) in LM, which produces heme for the photosynthetic machinery, and two Delta-aminolevulinic acid dehydratases involved in chlorophyll biosynthesis. In LM, we also detected the accumulation of Peptidyl-prolyl cis-trans isomerases (PPIases), similar to AtCYP38, and Protease Do-like 5, both involved in photosystem II repair^59^.

The enrichment analysis of DEPs detected in the comparisons RM *vs* RC and LM *vs* LC indicated that protein metabolism is one of the most up-regulated biological process in both roots and leaves of M plants (Fig. S6). In addition, transcriptomic and proteomic results revealed modulation of AA metabolism in response to the AM fungus in both roots and leaves (Figs S5 and S6). To confirm these data, free AA content was measured by LC-MS/MS analysis in both organs from C and M plants (Table 3). We observed in RM a significant increase in phenylalanine (Phe) and threonine, as expected by the down-regulation of lactoylglutathione lyase, which is involved in threonine degradation. In LM *vs* LC, we found an increase in Phe and tyrosine and a decrease in threonine, valine, serine and tryptophan. The decreased tryptophan might be related to the formation of N-benzoylanthranilate (involved in phytoalexin biosynthesis), as indicated by the down-regulation of anthranilate phosphoribosyl transferase and the increase in anthranilate N-benzoyltransferase protein 1. Significant decreases in glutamine, lysine and arginine were also detected in both RM and LM samples.

**Table 3:** Changes in free amino acids content.

	50 dpi		63 dpi
	RM/RC	LM/LC	LMX/LCX
*Aspartic acid*	1.34	0.96	0.89
*Alanine*	nd	nd	**2**.57**
*Arginine*	2	0.88	0.77
*Asparagine*	nd	**0**.72**	**3**.13*
*Cystine*	0.99	0.97	1.66
*Citrulline*	0.93	1.16	**0**.46*
*Ethanolamine*	0.99	1.07	1.47
*Phenylalanine*	**1**.19*	**1**.49*	**2**.06*
*GABA*	0.72	1.23	**1**.52*
*Glycine*	nd	nd	**2**.04*
*Glutamine*	0.68	**0**.52**	**1**.35*
*Histidine*	1.11	0.75	**1**.80**
*Leucine*	1.62	0.7	**1**.35**
*Lysine*	**0**.46*	**0**.42**	1.06
*Methionine*	1	1.05	1.61
*Ornithine*	1	0.96	**2**.83*
*Pea*	nd	nd	1.27
*Proline*	1.74	0.76	1.05
*Serine*	1.15	**0**.58**	**2**.25**
*Tyrosine*	1.32	**1**.47*	**1**.45*
*Treonine*	**1**.50*	**0**.74*	1.62
*Tryptophan*	1.27	**0**.46**	1.36
*Valine*	1.11	**0**.67*	**2**.31*

Values represent the amino acid content ratio between roots and leaves of control (C) and mycorrhizal (M) plants collected 50 days post AM fungus inoculation. LMX/LCX column represent the content ratio between leaves of control and mycorrhizal plants upon pathogen infection (CX and MX) at 14 days post *X*. *translucens* infection (corresponding to 63 days post AM fungus inoculation) (two-tailed *t* test; **P < 0.01 and *P < 0.05).

Transcriptomic and proteomic data sets provide strong evidence that AM fungi enhance primary metabolism in wheat, leading to corresponding changes in sugar, lipid and AA pathways. These dynamic changes in roots are mirrored by increased photosynthetic activity in leaves.

#### Phytohormone regulation

Both transcriptional and proteomic analyses highlight the regulation of genes and proteins involved in phytohormone pathways. When M plants were compared with C plants, several genes involved in auxin metabolism and transport were affected (Table S12). Accumulation of transcripts for *Auxin-responsive GH3-like*, *Auxin efflux carrier component 1*, *Auxin transporter-like protein* and (*SAUR*)-*like* genes was observed in leaves of M plants. By contrast, the genes and proteins induced in M roots were mostly involved in auxin homeostasis; these included IAA-AA conjugate hydrolase and indole-3-acetic acid-amido synthetase.

Since phytohormones can mediate plant immune responses and resistance induction^60^, we monitored the expression profiles of genes involved in Ethylene (ET), Salicylic Acid (SA), Jasmonic Acid (JA) and ABA metabolism. As observed in the shoots of mycorrhizal tomato plants^14,16^, among the most up-regulated genes we detected in LM several enzymes involved in ET biosynthesis: *ACC synthase 1* (*S-adenosyl-L-methionine methylthioadenosine-lyase 1-*, *1-aminocyclopropane-1-carboxylate oxidase; ACC oxidase 1* and *ACC oxidase 3*). Accordingly, in the RM sample, GO analysis showed the enrichment of DEPs involved in methionine and S-adenosylmethionine metabolism, both precursors in ET biosynthesis.

Several lipoxygenase (LOX) genes and proteins, and 12-oxophytodienoate reductase 1, were modulated in both organs of M plants. LOXs are crucial for lipid peroxidation processes and contribute to JA (through the 13-LOX pathway) and oxylipin biosynthesis, which play a pivotal role as signalling and protective molecules in plants responding to biotic stress^61^. In addition, *allene oxide synthase* (*AOS4*) and two *AOS2* genes, which catalyse the first step in the biosynthesis of JA, were highly expressed only in leaves of M plants.

Comparison of leaves and roots of M and C plants found that genes involved in SA biosynthesis or response were not differentially expressed. While 9-cis-epoxycarotenoid dioxygenase (NCED), involved in de novo ABA biosynthesis, and several genes involved in ABA catabolism (e.g., ABA 8′-hydroxylase and ABA-responsive protein-related) were up-regulated in both organs.

Taken as a whole, the dynamics of wheat phytohormone levels following *F*. *mosseae* inoculation confirms the scenario already described for other AM-host plant interactions^62,63^.

#### Local and systemic induction of defence genes and proteins in mycorrhizal plants

Although several studies have reported that defence reactions are activated in roots during the establishment of AM symbiosis, few have investigated such responses at the systemic level^4^. Analysis of the over-represented GO categories, detected in the comparison RM *vs* RC, highlighted the strong activation of genes involved in oxidation-reduction processes (Fig. S5). Also at the proteomic level, some ROS-scavenging enzymes, such as glutathione S-transferase (GST), ascorbate peroxidase, catalase, peroxiredoxin IIF, glutaredoxin and gamma-glutamyltranspeptidase 1, were differentially expressed in RM *vs* RC. M roots showed a significant up-regulation of some proteins involved in plant defence, including three acidic endochitinases, elicitor responsive gene 3, hypersensitive-induced reaction protein 3 and the cysteine-rich receptor-like protein kinases, Crk 25 and Crk6. *crk25* mutants are less resistant to pathogen infection^64^, while Crk6 is required for *X*. *oryzae-* and BTH-induced immune responses^65^. Several Germin-like genes and proteins (GLPs) were up-regulated in RM. GLPs, which are involved in defence responses, such as the oxidative burst, are up-regulated in roots following mycorrhizal colonisation^10,12,37,66^.

In the comparison LM *vs* LC, we identified 29 novel genes as exclusively expressed in the leaves of M plants and which are known to be involved in responses to biotic stress. These include a pathogenesis-related protein *PR-1*, three barley mildew resistance locus o (*MLO*) genes and putative homologs of *Resistance to Pseudomonas syringae* pv. maculicola 1 (*RPM1*) protein (Table S13). We also identified the induction of *Riboflavin synthase-like* (*RS*), an enzyme involved in the last steps of riboflavin biosynthesis. Riboflavin has been related to defence priming in *Arabidopsis*^67^, and it was also found to be over-accumulated in leaves of M tomato plants following *Botrytis cinerea* infection, suggesting its involvement in MIR^68^. Systemic responses were confirmed by proteomic analysis of LM *vs* LC, where an increase in some proteins involved in response to biotic stimuli, such as chitinase, PR protein, GLP and phenylalanine ammonia-lyase, was detected. Some enzymes involved in cell wall remodelling accumulated in the LM sample (*i*.*e*. beta-D-xylosidases 6 and 7, beta glucanase) and the non-specific lipid-transfer protein LPT142 which might play a role in wax or cutin deposition and in intracellular signal transduction. Finally, we found an increase in two PPIases that play a role in plant responses to heat, salinity and oxidative stress. Recent studies demonstrate that PPIases participate in responses to biotic stress in several plants, including wheat^69^.

When compared with their respective controls (LC and LCX), leaf protein profile 4A in both the LM and LMX samples exhibited induction of several proteins putatively involved in plant defence. Among them, we found W5ALA0, a protein highly homologous to ERDJ3B in *Arabidopsis*, involved in regulating protein folding in the endoplasmic reticulum lumen and which is necessary for pathogen-associated molecular pattern (PAMP)-triggered immunity^70^; W5B9I7, a protein showing 80% identity with NAA10 in *Arabidopsis*, and which is involved in plant immunity by maintaining homeostasis of the immune receptors SNC1 and RPM1^71^; and the Cell Division Cycle 5-like protein (CDC5) that, in addition to its function during mitosis, is also a core component of protein complexes that positively regulate defence responses through splicing and small RNA processing^72^.

In conclusion, mycorrhizal symbiosis leads to the activation of many defence-related genes not only in roots, but also in leaves of wheat. Taken as a whole, these genetic determinants, some never reported previously in an AM symbiosis, could predispose wheat to priming.

### Transcriptomic and proteomic changes induced by a leaf pathogen in wheat leaves and roots in the presence of an AM fungus

Our results (Fig. 2A,B) revealed that the AM fungus exerted a bio-protective effect against the wheat leaf pathogen *X*. *translucens*. Although wheat responses to this pathogen have already been investigated^73^, here we explored whether the pathogen triggered changes in transcript and proteomic profiles of leaves and roots in M plants, with special attention to genes related to pathogen resistance. For the “omics” experiment, we inoculated leaves of C and M plants with *X*. *translucens* (49 days following inoculation with the AM fungus) and collected the infiltrated zones and roots after 24 h. Global transcriptional and proteomic analyses were performed on both organs using RNA-seq and LC-MS/MS, respectively.

**Figure 6:**
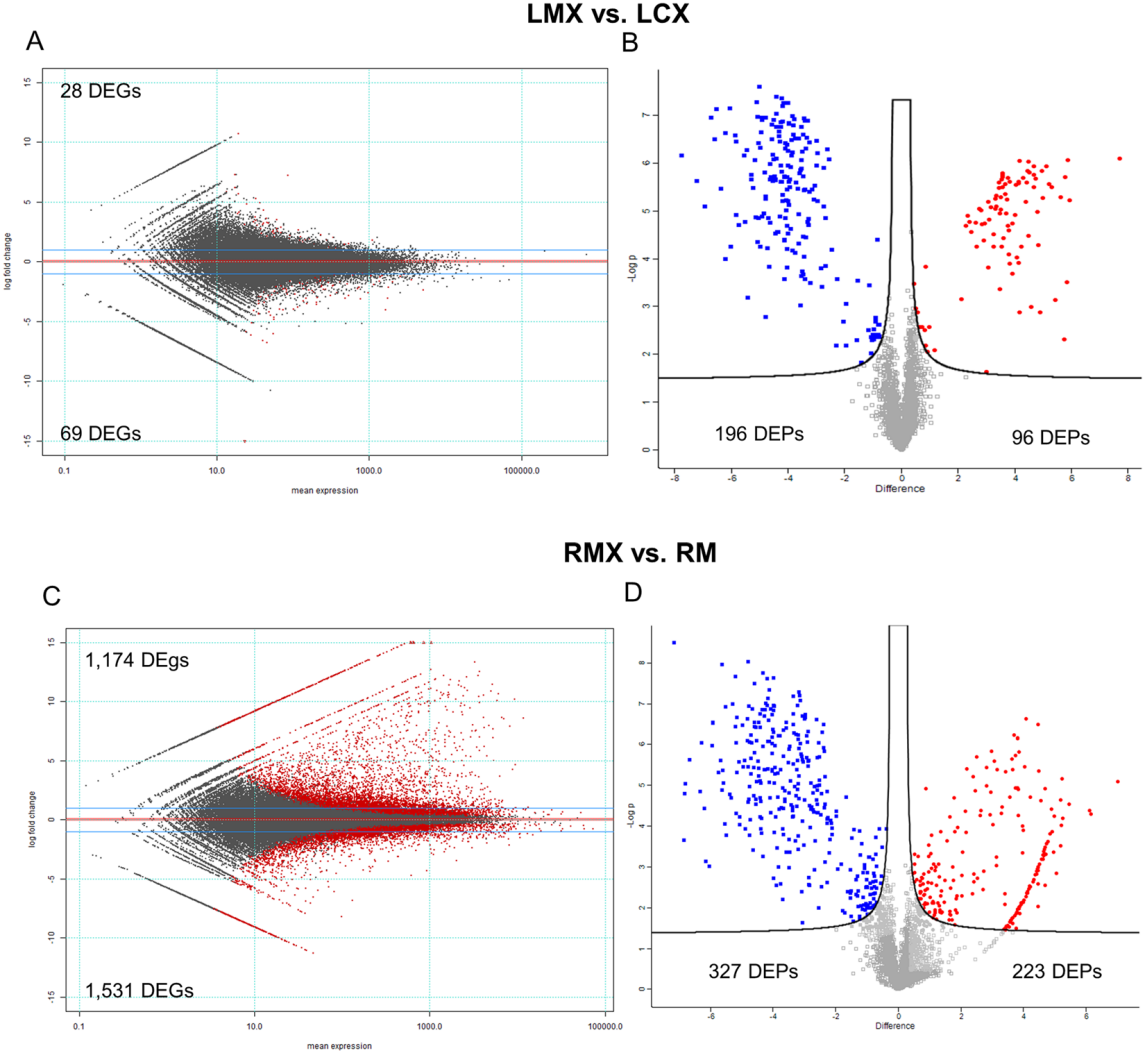
Global overview of the transcriptional and proteomic changes in the two organs (leaves -L and roots- R) following pathogen infection in control wheat plants (CX) and in mycorrhizal plants (MX). Mean expression versus log2 fold change plots (MA-plots, left side; Volcano plot, right side) were computed for these comparisons: LMX *vs* LCX (**A**,**B**), RMX *vs* RM (**C**,D). Called DEGs (**A**,**C**) and DEPs (**B**,**D**) (FDR 0.05) are plotted in color.

To gain an overview of the differentially expressed transcripts and proteins, we analysed the DEGs and DEPs obtained comparing leaves of C and M plants and root of M plants following pathogen infection (LMX *vs* LCX; RMX *vs* RM). In leaves, we detected 28 up- and 69 down-regulated genes (Table S14), and 96 up- and 196 down-regulated proteins (Table S15) (Fig. 6A–D). Omics data revealed an unexpected metabolic repression when the pathogen was inoculated on mycorrhizal plants. In this LMX *vs* LCX comparison, no over-represented biological process GO terms were identified at the transcriptomic level; however in LMX plants, the genes and proteins repressed during pathogen infection are mainly involved in AA metabolism (i.e., 3-deoxy-7-phosphoheptulonate synthase, Aspartate aminotransferase; asparagine synthetase, glutamine-dependent asparagine synthetase) (Fig. S8a), and defence responses (i.e., PR proteins, GSTs, defensin genes). The core set of 29 genes induced exclusively in LM plants and involved in responses to biotic stress (Table S13) was not differentially expressed in response of the pathogen (LMX *vs* LM, LMX *vs* LCX and LCX *vs* LC). This expression profile probably reflects the general down-regulation of plant metabolism imposed by the pathogen rather than any active role in hampering pathogen infection (Fig. 2).

The most up-regulated gene in LMX *vs* LCX was *premnaspirodiene oxygenase*, a cytochrome P450 enzyme, which functions in heme-iron binding and possesses oxidoreductase activity. The translated protein shared 80% identity with OsCYP71 from rice. *OsCYP71* is involved in early responses against *Xanthomonas oryzae pv*. *oryzae* (*Xoo*), and *OsCYP71-*overexpression lines confer resistance to *Xoo*^74^. Interestingly, this transcript was strongly down-regulated in LCX *vs* LC^73^. Another cytochrome P450 monooxygenase (CYP99A2), a precursor of the diterpenoid phytoalexins momilactones A and B, was also strongly induced in LMX *vs* LCX. Momilactones have been well characterised, and their antimicrobial activity against fungal pathogens has been reported^75,76^. In addition, OsCYP99A2 is involved in benzothiadiazole (BTH)-mediated priming in rice leaves^77^.

Nutrient availability has a strong impact on plant defence^78^, and nutrient uptake is a major factor in AM symbiosis^79^. In agreement with this statement, in the LMX *vs* LCX comparison, we identified the up-regulation of nitrate/chlorate transporters (NTRs). In addition, mineral content analysis revealed higher K (LMX/LCX: 1.18) and Cu (LMX/LCX: 1.9) content in LMX *vs* LCX after 14 days post pathogen infection.

Leaf protein profile 6A (Fig. 4) shows the exclusive induction in LMX of several proteins involved in plant defence responses, including two glucanases and cinnamoyl-CoA reductase 2, which were suggested to participate in the hypersensitive response to *X*. *campestris*^80^. H3.3, a protein that regulates gene body DNA methylation, belongs to the same profile. H3.3 knockdown in *Arabidopsis* reduces transcription of genes responsive to environmental cues^81^. In addition, some proteins involved in cell redox homeostasis, such as glutathione peroxidase and PDIL5, were up-regulated in LMX *vs* LCX.

To evaluate the systemic impact of leaf pathogen infection on the root, we analysed the DEGs and DEPs obtained in the RMX *vs* RM comparison. We detected 1174 up- and 1531 down-regulated genes (Table S16), and 223 up- and 288 down-regulated proteins (Table S17).

As expected, all the putative homologs of the AM marker genes (i.e., PTs and AMT genes) identified in the RM *vs* RC comparison were also detected in the RMX treatment; there was even a slight up-regulation of the genes in RMX *vs* RM. This up-regulation could be related to the slight increase in fungal colonisation detected in RMX *vs* RM plants. Even after 14 dpi the mineral content in RMX was comparable to RM, with the exception of K where a decreased level was detected in RMX (RMX/RM: 0.66).

Analysis of over-represented GO categories among the DEGs detected in the comparison of RMX *vs* RM highlighted a statistically significant enrichment of genes involved in responses to stress, including L-phenylalanine catabolic process, response to chitin, response to wounding, detection of biotic stimuli and defence response to fungi and bacteria (Figs S8b and S9). The most up-regulated genes were as follows: a predicted protein (Traes_2AL_3A3918F92.1) that shares 85% identity with a predicted antimicrobial peptides (AMPs) 1 protein from *Brachypodium distachyon*, which is involved in non-specific host defence^82^, and three PR proteins. A huge perturbation of genes belonging to the oxidation-reduction process category (98 up- and 88 down-regulated) was also observed.

Proteomic analysis revealed a statistically significant enrichment of DEPs involved in protein folding and ATP synthesis. In addition, GSTs, peroxidases, acidic endochitinases and heat shock proteins were increased (Table S16).

**Figure 7:**
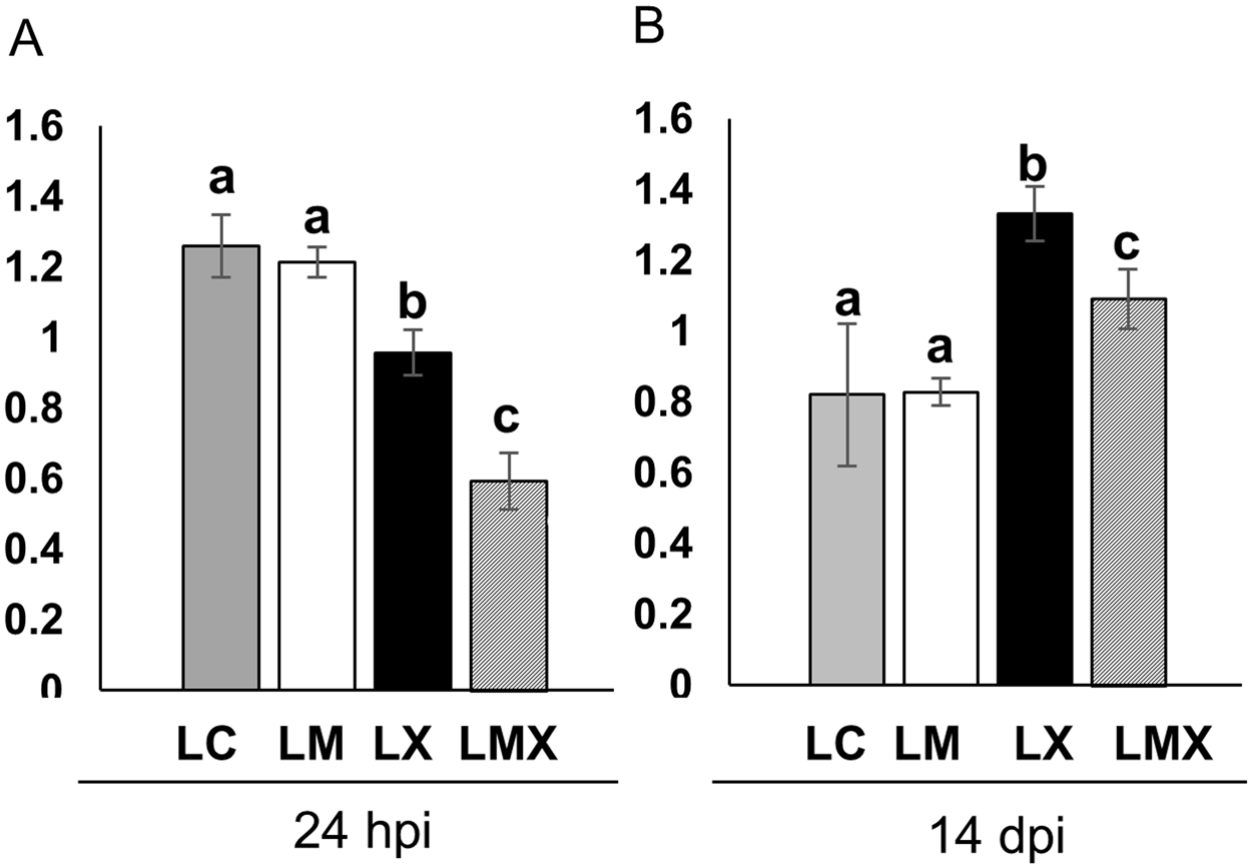
Protein carbonylation profiles in leaves of control and mycorrhizal wheat plants after *Xantomonas* infection at earlier (24 hpi) (**A**) and later (14 dpi) (**B**) time points. Each bar represents the protein carbonylation index, measured as ratio between the optical density (OD) obtained from the whole lane of the immunoblot and the OD of Coomassie stain. Data (means ± SD, n = 3) were subjected to one-way analysis of variance (ANOVA). Bars not accompanied by the same letters are significantly different at the 5% level using Tukey’s test.

To understand whether these responses were related to specific pathogen-induced oxidative damage, we compared the protein carbonylation profiles in leaves of M, C, CX and MX plants. Twenty-four hpi, both samples exposed to *X*. *translucens* (LCX and LMX) exhibited reduced protein carbonylation compared with non-infected plants (LC and LM). This suggests that the antioxidant defence systems in LCX and LMX were able to cope with the oxidative stress induced by pathogen infection, thereby avoiding irreversible protein oxidation in the earlier moment of the pathogen infection. However, LMX plants showed a stronger ROS-scavenging ability than LCX plants (Fig. 7A). At fourteen dpi, the level of oxidative damage in LCX and LMX plants was higher than in LM and LC plants. Again, the level of carbonylated proteins was consistently lower in LMX plants than LCX plants (Fig. 7B).

Overall, these results suggest that the AM fungus alleviates the symptoms caused by *X*. *translucens* through a broad down-regulation of transcript and protein levels, particularly evident in leaves. Here, a change in the level of protein oxidation, and a specific activation of genes involved in disease resistance and nutrient transport, suggests a specific MIR reaction.

Fourteen days after *X*. *translucens* inoculation, the content of most AAs was strongly reduced in LCX sample while the decrease was less pronounced in LMX (Table 2).

## Discussion

Although wheat is one of the most important sources for food, animal feed and industrial raw materials, limited attention has been paid to its response to AM fungi. This is mainly due to the difficulty in overcoming the challenges imposed by the large wheat genome, comprising three complete genomes, multiple gene duplications and extensive regions of suppressed recombination^19^. These genome features make traditional genetic tools, such as mutant availability and genetic transformation protocols, time-consuming and costly, making this host plant less suitable for the functional characterisation of the AM symbiosis.

### AM symbiosis leads to short- and long-term benefits in wheat

The high level of genetic diversity of wheat is mirrored by a diverse responsiveness to AM symbiosis when different genotypes are considered^83^ and by contrasting results in terms of perceived benefits (i.e., biomass and nutrient acquisition)^20,25,84^. Our greenhouse experiments revealed that all measured agronomic traits were higher in mycorrhizal wheat (cv. Chinese Spring) plants, including an improvement in nutrient and AA content of seeds. The higher levels of Mg, Zn and P that were detected in seeds suggest potential benefits to the offspring, such as faster growth, a better chance of surviving in mineral-deficient soils and, especially for crops, improved grain yield and quality^85–88^. Although increases in seed size, yield and germination rate have been observed in different plant species in symbiosis with AM fungi^89–91^ effects on wheat grain have received little attention. Our results demonstrate substantial changes in seed morphology and nutrient content, opening the way to further investigations of long-term benefits for offspring and of transgenerational mycorrhizal effects on progeny phenotype.

By determining genome-wide transcriptional changes during mycorrhizal establishment, DEGs involved in numerous processes in plants were identified. In both dicots and monocots, massive transcriptional changes occur during mycorrhizal establishment, and a core of AM symbiosis marker genes, considered the functional signature of the symbiosis, have been identified^14,37^. Focusing on rice, for which data sets from whole-genome transcript profiling of mycorrhizal roots are available^9,10,37^, we wondered whether the well-annotated rice genome could provide a track to decipher AM responses in wheat. Comparative analysis highlighted an extensive overlap in the transcriptional responses of rice and wheat mycorrhizal roots, allowing us to identify several AM marker genes in wheat roots. Among them, several nutrient transporter genes, including PTs and AMT, were identified, as confirmed by the increased content of several mineral nutrients in roots and leaves. Mg and Fe contents were lower in RM plants and higher in LM plants, suggesting a root to aboveground organ translocation. Considering that Fe and Mg are essential macronutrients involved in numerous physiological processes, including photosynthetic CO_2_ fixation, biomass production, grain yield and plant immunity^92–95^, it is tempting to speculate that AM symbiosis might also improve wheat development via Fe and Mg acquisition. These data support the concept that positive mycorrhizal growth responses arise largely from the increased uptake of growth-limiting nutrients^96^. Our work also reports a strong impact exerted by the AM fungus on primary metabolism and phytohormone regulation of the host plant at both the local and systemic level, suggesting that other metabolic pathways are involved in these growth responses.

Meyer and colleagues^97^ proposed that the increased biomass in *Arabidopsis* is orchestrated by a combination of metabolites that act synergistically. Among them, sugars such as sucrose, members of the TCA cycle and metabolites involved in membrane/phospholipid biosynthesis such as G3P are involved in the control of plant growth and development. Several wheat genes and proteins involved in these carbohydrate and lipid pathways were strongly induced in both RM and LM plants, suggesting that the increased biomass in AM-infected plants is related to the general mechanisms described for *A*. *thaliana*.

In conclusion, both greenhouse and growth chamber experiments demonstrated growth benefits to wheat following AM inoculation. The omics results confirm previous findings (e.g., improved expression of nutrient transporters), but also provide novel information: a complex pattern of local and systemic changes in gene expression with similarities and differences between roots and leaves, changes in nutrient and AA contents, and effects on seeds.

### At least two processes guarantee the bio-protection effect exerted by AM symbiosis against a leaf pathogen

The bio-protective role of AM symbiosis against crop pathogens has long been recognised on the basis of experimental data showing significant reductions in disease symptoms^8,32,33^. The molecular basis of such protective responses includes elicitation of defence-responsive genes, accumulation of defensive compounds at both local and systemic levels^8,12,14,26,98,99^ and modulation of defence-related hormones^4,26,34^.

In leaves of mycorrhizal rice plants, a two-step mechanism has been proposed to control the enhanced resistance against aboveground pathogens: a preliminary induction of defence mechanisms (priming) in the absence of the pathogen, followed by a faster and stronger activation of *PR* genes upon pathogen infection^8^. Our experiments revealed that, in wheat, the AM-induced bio-protective effect requires the integration of multiple mechanisms. As with other host plants, wheat roots activated a set of ROS-scavenging enzymes, as well as acidic endochitinases and PR proteins. We detected 29 defence-responsive genes (e.g., *PR proteins*, *RPM1*, *MLO*) exclusively regulated in the leaves of mycorrhizal wheat, as well as genes and proteins (e.g., *RS*, GLP, PAL) involved in the categories “response to biotic stimuli” and “plant immunity”. These data suggest that mycorrhizal wheat activates a *broad-spectrum defence* (*BSD*) response, where genes and proteins playing a regulatory role in the host immune system are activated. The differential regulation of genes involved in the biosynthesis of defence-related hormones (i.e., JA, ET and ABA)^60^, as well as changes in AAs, whose role in orchestrating plant-microbe interactions^100^ and the plant immune system^101^ is emerging, reveal that the *BSD* response is part of a larger reprogramming scenario induced by the presence of AM fungi in the roots. The main emerging question is to understand whether such a *BSD* response, which leads to a priming state, is sufficiently effective to provide protection against a leaf pathogen, i.e., to activate MIR^4,6^. Although some pathways systemically elicited by the AM fungus, and listed as a *BSD* response, were also maintained during interaction with the pathogen, our results reveal that, following pathogen infection, leaves of mycorrhizal plants strongly induce different defence genes and proteins that we identify as a *pathogen-specific defence* (*PSD*) response. Even if there was a general repression of many metabolic pathways, to counter pathogen attack, LMX plants accumulated transcripts and proteins involved in redox homeostasis, phytoalexin production and the hypersensitive response, all of which represent a *PSD* response. An example of such a *PSD* response is the elevated expression of two cytochrome P450 monooxigenase genes (*premnaspirodiene oxygenase* and *CYP99A2*) and two glucanases and a cinnamoyl-CoA reductase 2 proteins in LMX plants that have been reported in other plants to play a key role in hampering bacterial infection and to be involved in the priming response^74–77,80^. Changes in the abundance of carbonylated proteins, considered to be markers of oxidative stress, indicate that, at 14 dpi, when lesions are clearly visible on leaves, the presence of the AM fungus in the roots lightens the symptoms caused by the pathogen.

**Figure 8:**
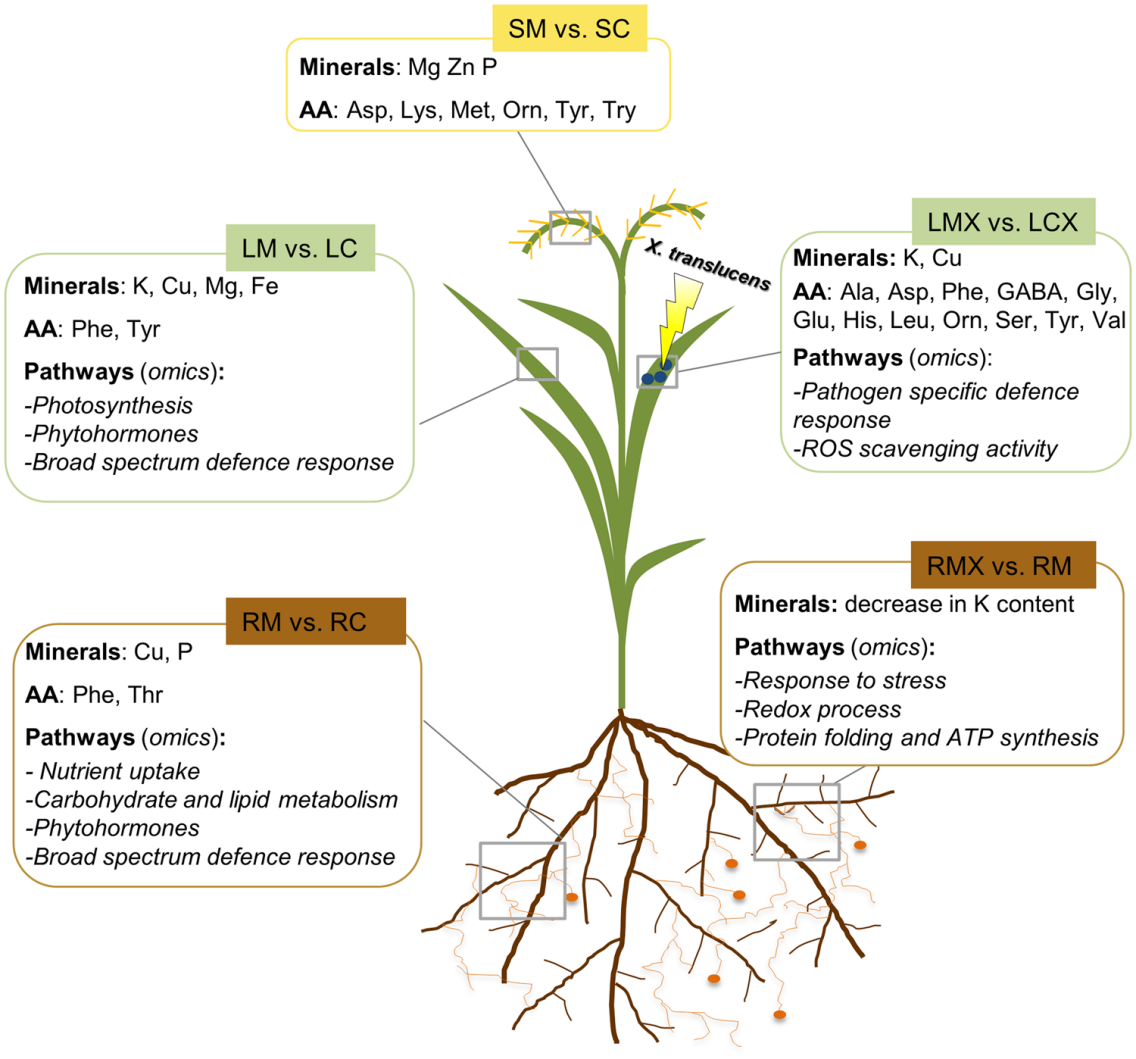
Schematic representation of local and systemic changes in wheat plants upon mycorrhizal symbiosis and following pathogen infection. SM: seeds of mycorrhizal plants; SC: seeds of control plants; LM: leaves of mycorrhizal plants; LC: leaves of control plants; RM: mycorrhizal roots; RC: control roots. LMX: leaves of mycorhizal plants infected by *X. translucens*; LCX: leaves of control plants infected *by X. translucens*; AA: amino acid.

In summary, deep omics analysis supported by metabolic data showed that AM symbiosis confers on wheat higher productivity and resistance to *X*. *translucens* infection (Fig. 8). The higher productivity is accompanied by local and systemic activation of pathways involved in nutrient uptake, primary metabolism and phytohormone regulation. These benefits seem to have long-term effects, since they are also effective on seeds. In addition to this large-scale metabolic reprogramming, we also identified defence-related pathways that have been described previously in roots of many other AM plants, as well as a novel core set of genes exclusive to leaves of mycorrhizal plants. While this represents a *broad-spectrum defence*, we also detected a second *pathogen-specific defence* response, which occurs following bacterial infection and promptly activates genes already identified as operational in resistance to *Xanthomonas*. Together with the regulation of phytohormones and AA biosynthesis, we suggest that this two-step response contributes to MIR.

In conclusion, we propose that the activation of a *BSD* response, occurring in roots and leaves of mycorrhizal plants, makes wheat ready to switch to a *PSD* response upon bacterial infection, leading eventually to effective protection against the invader.

## Material and Method

### Greenhouse trial

Two pot experiments were conducted during the 2015–2016 seasons in the greenhouse of Genomics Research Centre (Fiorenzuola, PC, Italy) to evaluate the effect of *Funneliformis mosseae* (BEG12; MycAgro Lab, France) on some wheat agronomic traits. The experiments had two treatments, mycorrhizal and non-mycorrhizal plants and were carried out in 15 × 15 × 20 cm square pots, filled with a 50:50 mixture of sterile sand and field soil. This soil, from the Fiorenzuola’s experimental farm located in Northern Italy (44°55′19.4″N 9°54′32.3″E), was sieved (2 mm) and dry treated at 100 °C × 1 hour ×3 times, during three consecutive days. The pasteurized soil resulted alkaline (pH 8.21), with the following characteristics: total carbonate 12.66%; inorganic carbon 15.19 g/kg; total carbon 28.1 g/kg; organic carbon 12.9 g/kg; organic matter 2.23%; total N 0.11%; C:N ratio 11.8; P2O5 35.4 mg/kg; cation exchange capacity 7.17 cmol(+)/kg. Before potting, all pots were washed with 0.1% HCl and deionized water and filled with 3.5 kg of soil. *F*. *mosseae* (formely *Glomus mosseae* BEG.12) obtained from MycAgro Lab. (Technopole Agro-Environement, Bretenière, France) was inoculated 2–3 cm below the planting holes in thirty replicated pots (10 propagules g-1 soil). Other thirty pots were not inoculated. Seeds of bread wheat (*Triticum aestivum* Desf.; cv Chinese Spring) were surface-sterilized and germinated on wet filter paper in Petri dishes for 5 days in the dark, at 20 °C. Five seedlings were transplanted into each pot and after 3 days the pots were thinned to three plants each. Plants were cultivated in greenhouse with natural light and temperature from January to July as randomised design. The plants were irrigated with tap water to keep substrate at 80% field capacity without any fertilization until full ripening. At the end of tillering stage the number of tillers/plant was recorded. At Z92 cereal growth staging scale^102^ all plants were harvested. Aboveground plant dry biomass and spike weights were recorded for each pot. The spikes were threshed and 1000-kernel weights were determined. Grains were scanned using a flatbed scanner (Epson Expression 10000XL) and the images were processed with the GrainScan software^103^ to determine grain area, length and width. The roots were recovered, thoroughly washed and used for DNA extraction. The presence of *F*. *mosseae* DNA was evaluated with quantitative real time-PCR analysis, using the *F*. *mosseae* BEG12 primers according to^104^.

### Plant growth condition, inoculation with AM fungus and the pathogenic bacterial strain

For standard laboratory conditions, *Triticum aestivum* cv. Chinese Spring seeds were surface sterilized and then germinated on filter paper wetted with distilled water. The seedlings were then transferred to pots with sterile quartz sand. Inoculation of *Funelliformis mosseae* (BEG.12; MycAgro Lab, France) were performed by mixing the inoculum with sterile quartz sand (30% v/v). Mycorrhizal and non-inoculated (control) plants were maintained under glasshouse conditions under cycles of 12 h of light at 21 °C and 50% relative humidity (RH) and 12 h of dark at 21 °C and 50% RH, watered twice a week with water, and once with a modified Long-Ashton solution containing a low phosphorous concentration (32 µM Na_2_HPO_4_*12H_2_O). After 49 days post AM fungal inoculation (dpi), a set of mycorrhizal and control plants were inoculated with *X*. *translucens* type strain CFBP 2054 (syn. ATCC 19319, DSM 18974, ICMP 5752, LMG 876, NCPPB 973; GeneBank acc. no. CAPJ01000000) as described by^73^. Controls were inoculated following the same procedure but with water. For the transcriptomic and proteomic (*omics*) experiments the infiltrated zone was collected 1 day post-inoculation (dpi) by pathogen when symptoms were not visible. While for phenotypic observation infected plants were left to grow and symptoms (water-soaked lesions) were scored 14 dpi. For the “omics” experiments, plants were harvested 50 dpi. Portions of the root system from each mycorrhizal plant were selected under stereomicroscope on the basis of the presence of external mycelium. These root portions were mixed and pooled together and then divided into two samples, one to assess the level of mycorrhizal formation (done over 80 cm of root per sample), and the other for RNA extraction. The mycorrhizal roots were stained with cotton blue and the level of mycorrhizal colonisation was assessed according to Trouvelot *et al*.^105^. Root and shoot fresh biomass of C and M plants were measured and evaluated with the analysis of variance. Parallel independent experiments were conducted to estimate the production, in the same conditions of previous trials. Spikes weight were measured separately in the mature plants at the end of their natural cycle. Two plants were pooled per replicate, with three biological replicates per treatment. Once pulverized in liquid nitrogen, each sample was split into two aliquots, one for protein extraction and one for RNA extraction.

### Nucleic acid extraction, cDNA synthesis and Real-time quantitative RT-PCR

Total genomic DNA was extracted from *F*. *mosseae* sporocarps as described in Fiorilli *et al*.^14^, and from *T*. *aestivum* shoots using the DNeasy Plant Mini Kit (Qiagen, Hilden, Germany), according to the manufacturer’s instructions. Plant and fungal genomic DNAs were used to test each primer pair designed for real-time PCR to exclude cross-hybridization.

Total RNA was extracted from *T*. *aestivum* shoots and roots of M, C plants, as well as control and mycorrhizal plants infected by the bacterial pathogen *X*. *translucens* (CX and MX) as described by Garcia-Seco *et al*.^73^. After DNase treatment and confirmation of RNA integrity using Experion™ Automated Electrophoresis System, and gel electrophoresis, the total RNA was used for mRNA preparation, fragmentation and cDNA synthesis.

Quantitative Real-time was used to measure the expression of nine genes shown to be differentially regulated by RNA-Seq. Single-strand cDNA was obtained as described in Vallino *et al*.^48^. Three biological replicates were conducted for each condition. Quantitative real time PCR experiments and data analysis were carried out as described in Vallino *et al*.^48^. The primer names and corresponding sequences are listed in Table S1.

### RNA-seq transcriptome sequencing

The preparation of mRNA libraries was done by TrueSeq Stranded mRNA Sample (Illumina) Preparation. The library quality validation (fragment concentration and length) was performed by Fragment Analyzer using the standard and high sensitivity DNA kits (Agilent Technologies, Santa Clara, CA) and by qPCR (LightCycler 480; Roche Diagnostics, Meylan, France). RNA-seq library preparation and sequencing was carried out by MGX-Montpellier GenomiX platform using a HiSeq. 2500 instrument (Illumina Inc., San Diego, CA). Six libraries were sequenced per lane (around 30 millions reads per replicate), with 100 nucleotides sequence length per read.

### Bioinformatic analysis of transcriptome data

Mapping and alignment of sequence reads was done with Burrows-Wheeler transform (BWA-MEM algorithm) using the BWA-MEM algorithm. Reads were subsequently aligned to *T*. *aestivum* coding sequences (CDS) (IWGSC1.0 + popseq. 29) and non-coding RNA (ncRNA). Subsequently, sequence counts were collected by Samtools idxstats filtering of bam alignment files for reads with MAPQ ≥ 3. Differentially expressed genes (DEG) were called via DESeq. 2 1.10.0 Bioconductor package using local fit and betaPrior parameter set to False. Independent filtering was enabled^106^. A false discovery rate threshold of 0.05 was set for DEG calling. Sample clustering and principal component analyses were performed upon variance stabilizating transformation of expression data. Transcripts were considered differentially expressed when the adjusted FDR values are below 0.05 and the logarithmic fold change over 0.5 or when the logarithmic fold change was over 2. The transcriptomics data related to roots and leaves of mycorrhizal plant and roots and leaves of mycorrhizal plants infected by X. *translucens* have been deposited in the European Bioinformatics Institute (EMBL-EBI) ArrayExpress database (https://www.ebi.ac.uk/arrayexpress) with the dataset identifier E-MTAB-5898, while the transcriptomic data from control plants and control plants infected by *X*. *translucens* with the dataset indentifier E-MTAB-5891^73^. Unless otherwise stated, further graphical outputs were generated with custom R and Python scripts.

### Protein extraction and Liquid Chromatography-Mass Spectrometry (LC-MS/MS) analysis

Total proteins were extracted from *T*. *aestivum* shoots and roots of M, C, CX and MX plants as described by Garcia-Seco *et al*.^73^.

MS analysis was performed on a QExactive mass spectrometer coupled to a nano EasyLC 1000 (Thermo Fisher Scientific Inc., Waltham, MA). Solvent composition at the two channels was 0.1% formic acid for channel A and 0.1% formic acid, 99.9% acetonitrile for channel B. For each sample, 4 μL of peptides were loaded on a self-made column (75 μm × 150 mm) packed with reverse-phase C18 material (ReproSil-Pur 120 C18-AQ, 1.9 μm; Dr. Maisch GmbH, Ammerbuch, Germany) and eluted at a flow rate of 300 nL/min by a gradient from 2 to 35% B in 80 min, 47% B in 4 min and 98% B in 4 min. Samples were acquired in a randomized order. The mass spectrometer was operated in data-dependent mode (DDA), acquiring a full-scan MS spectra (300–1700 m/z) at a resolution of 70,000 at 200 m/z after accumulation to a target value of 3,000,000, followed by HCD (higher-energy collision dissociation) fragmentation on the twelve most intense signals per cycle. HCD spectra were acquired at a resolution of 35,000 using a normalized collision energy of 25 and a maximum injection time of 120 ms. The automatic gain control (AGC) was set to 50,000 ions. Charge state screening was enabled and singly and unassigned charge states were rejected. Only precursors with an intensity above 8300 were selected for MS/MS (2% underfill ratio). Precursor masses previously selected for MS/MS measurement were excluded from further selection for 30 s, and the exclusion window was set at 10 ppm. The samples were acquired using internal lock mass calibration on m/z 371.1010 and 445.1200.

### Proteomic data processing and bioinformatic analysis

Mass spectrometer raw files were analyzed by MaxQuant (version 1.5.3.28 and 1.5.3.30) with the match between runs (matching time window of 2 min) and label free quantification (LFQ) options selected. Tandem MS spectra were searched against UniProt *T*. *aestivum* (Version 2015–10, 100,800 entries), Uniprot *Rhizophagus irregularis* (Version 2015–10, 29,847) and Uniprot *Xanthomonas translucens* (Version 2015–10, 14,378 entries). Trypsin/P was chosen as the protease, cysteine carbamidomethylation was set as fixed modification, and oxidation of methionine and acetylation of the N-terminal as variable modifications. Peptide tolerance was set to 4.5 ppm, while MS/MS tolerance was set to 0.5 Da. Peptide-spectrum matches (PSMs) and proteins were validated with 1% FDR. Only PSMs with a minimum length of 7 amino acids were kept.

The raw data was first processed by Perseus (MaxQuant package) and the incorrected protein identifications (contaminants, decoy and “only identified by site” entries) were removed from the main data frame. LFQ intensities were Log2 transformed. Only protein groups detected in almost two of three biological replicate samples sharing the same treatment and tissue were considered unambiguously identified and were used for assessment of significant change. Missing value were imputed using the *R* package ‘imputeLCMD’ (https://cran.rproject.org/web/packages/imputeLCMD/). The experimental quality was checked using the multi-scatter plots tool for the analysis of Pearson correlation between the samples. To determine the differentially expressed proteins (DEPs) among considered conditions, we performed a multiple sample test using Anova with a permutation-based false discovery rate (FDR) cutoff of 0.01.

For the annotation of the unknown proteins a blast search was made against the Uniprot database viridiplantae (Version 2015–10, 3398870 entries), taking the first hit with a valid annotation. For the GO analysis of DEPs we used agriGO v2.0 (Tian *et al*. 2017) and for the cluster analysis, we used the “Profile plot” tool of Perseus program.

### Aminoacid analysis

HPLC-grade water, HPLC-grade methanol (MeOH), formic acid, aspartic acid (Asp), asparagine (Asn), glutamine (Gln), glutamic acid (Glu), serine (Ser), threonine (Thr), cystine (Cys), alanine (Ala), proline (Pro), valine (Val), methionine (Met), tyrosine (Tyr), leucine (Leu), phenylalanine (Phe), tryptophan (Trp), lysine (Lys), histidine (His) arginine (Arg) ornithine (Orn), pipecolic acid (Pip), citrulline (Cit), GABA and two deuterated internal standards (L-Phenyl-d5-alanine and L-*alanine* ^*15*^N) were purchased from Sigma–Aldrich Co. (Dorset, UK).

Stock solutions for all compounds were prepared in distilled water (10 mM). A working mixture (1 mM of each aminoacid, AA) was prepared and used for the calibration (range 2–15 µM).

For the AA extraction, all samples were lyophilized and 0.1 g of each sample was re-suspended in 10 mL of 0.1% (v/v) formic acid in water/methanol (50:50). 10 μl of 10 mM deuterated internal standards were added. The mixture was then vortexed for 4 h in the dark, sonicated for 15′, centrifuged and the supernatant was collected.

The HPLC analysis was performed in a Finningan Surveyor MS plus HPLC system (Thermo Electron Corporation, CA, USA). Separation was achieved using C18 column (Phenomenex, synergi 4 u fusion-RP 80a 150 × 2.00 mm). The mobile phase was composed of (A) water with 0.1% (v/v) formic acid and (B) methanol/water (50:50) 0.1% (v/v) formic acid with flow rate 150 µL/min; gradient 0–3.0 min/2% (v/v) B, 3–16 min/2–50% (v/v) B. For the mass spectrometry quantification, a Finningan LXQ linear ion trap mass spectrometer, equipped with an ESI ion source (Thermo Electron Corporation, CA, USA), was used. The analyses were done in positive (spray voltage 4,5 kV, capillary temperature 270 °C) and in the multiple reaction monitoring (MRM) mode. The optimization of collision energy for each substance, the tuning parameters and the choice of fragments to confirm the identity of target compounds were done in continuous flow mode, by using standard solution at concentration of 5 μM (Table S2). The linearity of the method was considered adequate when square correlation coefficient (R^2^) was higher than 0.98, based on peak area. The limits of detection (LOD) and quantification (LOQ) were fixed at 1 µM and at 2 µM, respectively.

### Minerals content analysis

All frozen samples, were lyophilized, dried in an oven at 60 °C for 8 h and then mineralized with 1 mL of HCl and 1 mL of hydrogen peroxide (H_2_O_2_). Samples were reconstituted in 1% HNO_3_ in Milli-Q water. Blanks were made with the same solvents and chemicals employed in the treatment and digestion of the samples, or with just 1% HNO_3_ in Milli-Q water. Calibration standard solutions were prepared from 1000 mgLl^−1^ standard solutions of Mg, Fe, Cu, Zn and K (Baker Instra-Analyzed). The determination of minerals was performed on a Thermo Fisher Solaar M6 atomic absorption spectrometer. Ca, Mg, Fe, Zn, K were determined at ppm levels by flame atomic absorption spectrometry (FAAS) with deuterium lamp background correction; Cu was determined at ppb levels by graphite furnace atomic absorption spectrometry (GFAAS) and Zeeman background correction. All parameters such as the wavelength and the bandpass were set according to the recommendations of the instrument Cookbook.

The phosphorous content determination was performed as reported by Chen and Toribara^107^.

#### Detection of carbonylated proteins

Carbonylated proteins were detected in all samples collected at 1 dpi and 14 dpi, as described previously^71^. Protein carbonylation index (arbitrary units) was measured as ratio between the optical density (OD) obtained from the whole lane of the immunoblot and the OD of Coomassie stain. For each set (Leave and Roots) data (means ± SD, n = 4) were subjected to one-way analysis of variance (ANOVA) and post-hoc Tukey’s test.

## Electronic supplementary material

Supplementary figures

Supplementary tables
