# Supplementary material for: Omics approaches revealed how arbuscular mycorrhizal symbiosis enhances yield and resistance to leaf pathogen in wheat
Source: Sci Rep. 2018 Jun 25;8:9625. doi: 10.1038/s41598-018-27622-8 (PMC6018116; doi:10.1038/s41598-018-27622-8)
Supplement: Supplementary file 1 — Supplementary figures [file 41598_2018_27622_MOESM1_ESM.pdf]

Omics approaches revealed how arbuscular mycorrhizal symbiosis enhances yield and resistance to leaf pathogen in wheat

Fiorilli Valentina, Vannini Candida, Ortolani Francesca, Garcia-Seco Daniel, Chiapello Marco, Novero Mara, Domingo Guido, Terzi Valeria, Morcia Caterina, Bagnaresi Paolo, Moulin Lionel, Bracale Marcella, Bonfante Paola.

Supporting Material legends

Fig. S1: Systemic effect on wheat seeds nutrient and amino acid composition exerted by *F. mosseae*. A) The picture shows the greenhouse seeds collected from control (C) and mycorrhizal plants (M). B) Changes in the mineral content in greenhouse seeds. Ratios of the mean values of 3 replicate samples (μg/g dry weight; two-tailed t test $*P < 0.05$) are given. C) Changes in levels of free amino acids in roots and shoots samples (two-tailed t test $*P < 0.05$)

Fig. S2: Venn diagrams of control and AM fungal-modulated genes and proteins (DEGs and DEPs) in roots and leaves.
Venn diagram showing the relationships between DEPs and DEGs in response to arbuscular mycorrhizal (AM) symbiosis in the roots (A) and leaves (B). The number of DEPs and DEGs commonly expressed is shown in the overlapping portion. Functional distribution of the common DEGs and DEPs in the roots (C) and leaves (D) of wheat colonized by *F. mosseae*; genes and proteins were grouped in comprehensive groups on the basis of their gene ontology (GO) annotation.

Fig. S3: The evolutionary history of the mycorrhiza-induced phosphate transporter (PT) genes. The amino acid sequences were aligned using Clustal Omega (Sievers et al., 2011) and the LG + G substitution model was selected under the Akaike criterion using ProtTest 3.4.2 (Darriba et al., 2011). Maximum likelihood was estimated with PhyML 3.0 (Guindon and Gascuel, 2003) and the resulting neighbour joining tree was visualized with FigTree (http://tree.bio.ed.ac.uk/software/figtree/). The tree is drawn to scale, with branch lengths in the same units as those of the evolutionary distances used to infer the phylogenetic tree. Abbreviations for plant species: Le, *Licopersicum esculentum*; Lj, *L. japonicus*; Mt, *Medicago truncatula*, Os, *O. sativa,* Ta*, Triticum aestivum*. Asteric indicate the ID of a non-annotated PT gene of *Triticum aestivum.*

Fig. S4: Relative gene expression of ten differentially expressed genes. qRT-PCR analysis was done on genes selected from DEGs identified in the comparison between control (RC) and mycorrhizal (RM) roots. The y-axis represents relative expression. Values are means of three replicates. Error bars represent standard deviation. Asterisks indicates significant difference at p-value < 0.01, according to the one-way ANOVA with Tukey's post-hoc test.

Omics approaches revealed how arbuscular mycorrhizal symbiosis enhances yield and resistance to leaf pathogen in wheat

Fiorilli Valentina, Vannini Candida, Ortolani Francesca, Garcia-Seco Daniel, Chiapello Marco, Novero Mara, Domingo Guido, Terzi Valeria, Morcia Caterina, Bagnaresi Paolo, Moulin Lionel, Bracale Marcella, Bonfante Paola.

Supporting Material legends

Fig. S5: Plots illustrating enriched GO terms specific for RM vs RC contrast. The enriched GO terms (y axis labels; FDR threshold for GO enrichment ≤ 0.1) associated to DEGs in the contrast are shown. Reference set was defined as all genes with expression above 0.1 RPKM. X-axis displays the reciprocal of Enrichment p value as calculated with the Goseq R package. Enriched GO terms belong to the category biological process.

Fig. S6: Plots illustrating enriched GO terms specific for LM vs LC contrast. The enriched GO terms (y axis labels; FDR threshold for GO enrichment ≤ 0.1) associated to DEGs in the contrast are shown. Reference set was defined as all genes with expression above 0.1 RPKM. X-axis displays the reciprocal of Enrichment p value as calculated with the Goseq R package. Enriched GO terms belong to the category biological process.

Fig. S7: Single enrichment analysis of protein GO terms specific for RM vs RC and LM vs LC contrasts. The enriched GO terms (FDR threshold for GO enrichment ≤ 0.05) associated to DEPs in the contrasts are shown. Reference set was defined as Uniprot 2016 database. Enriched GO terms belong to biological process term.

Fig. S7: Single enrichment analysis of protein GO terms specific for RM vs RC and LM vs LC contrasts. The enriched GO terms (FDR threshold for GO enrichment ≤ 0.05) associated to DEPs in the contrasts are shown. Reference set was defined as Uniprot 2016 database. Enriched GO terms belong to biological process term.

Fig. S9: Plots illustrating enriched GO terms specific for RMX vs RM contrast. The enriched GO terms (y axis labels; FDR threshold for GO enrichment ≤ 0.1) associated to DEGs in the contrast are shown. Reference set was defined as all genes with expression above 0.1 RPKM. X-axis displays the reciprocal of Enrichment p value as calculated with the Goseq R package. Enriched GO terms belong to biological process term.

Omics approaches revealed how arbuscular mycorrhizal symbiosis enhances yield and resistance to leaf pathogen in wheat

Fiorilli Valentina, Vannini Candida, Ortolani Francesca, Garcia-Seco Daniel, Chiapello Marco, Novero Mara, Domingo Guido, Terzi Valeria, Morcia Caterina, Bagnaresi Paolo, Moulin Lionel, Bracale Marcella, Bonfante Paola.

A

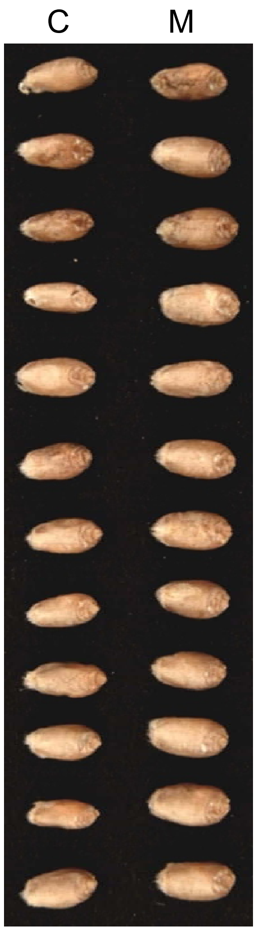

B

Mineral	M/C
Ca	1.03
Mg	**1.39***
K	1.04
Fe	0.82
Zn	**1.32***
Cu	1.08
P	**1.19***

C

AA	M/C
Aspartic acid	0.82
Glutamic acid	0.73
Alanine	0.85
Arginine	1.07
Asparagine	**1.29***
Cystine	1.22
Citrulline	1.15
Phenylalanine	1.37
GABA	1.17
Glycine	1.12
Glutamine	0.88
Histidine	1.32
Leucine	0.84
Lysine	**0.58***
Metionine	**1.31***
Ornitine	**1.30***
Proline	0.82
Serine	0.89
Tyrosine	**1.35***
Threonine	0.96
Tryptophan	**1.92***
Valine	0.92
AA tot	1.14

Fig. S1. Systemic effect on wheat seeds nutrient and amino acid composition exerted by *F. mosseae*.

Omics approaches revealed how arbuscular mycorrhizal symbiosis enhances yield and resistance to leaf pathogen in wheat

Fiorilli Valentina, Vannini Candida, Ortolani Francesca, Garcia-Seco Daniel, Chiapello Marco, Novero Mara, Domingo Guido, Terzi Valeria, Morcia Caterina, Bagnaresi Paolo, Moulin Lionel, Bracale Marcella, Bonfante Paola.

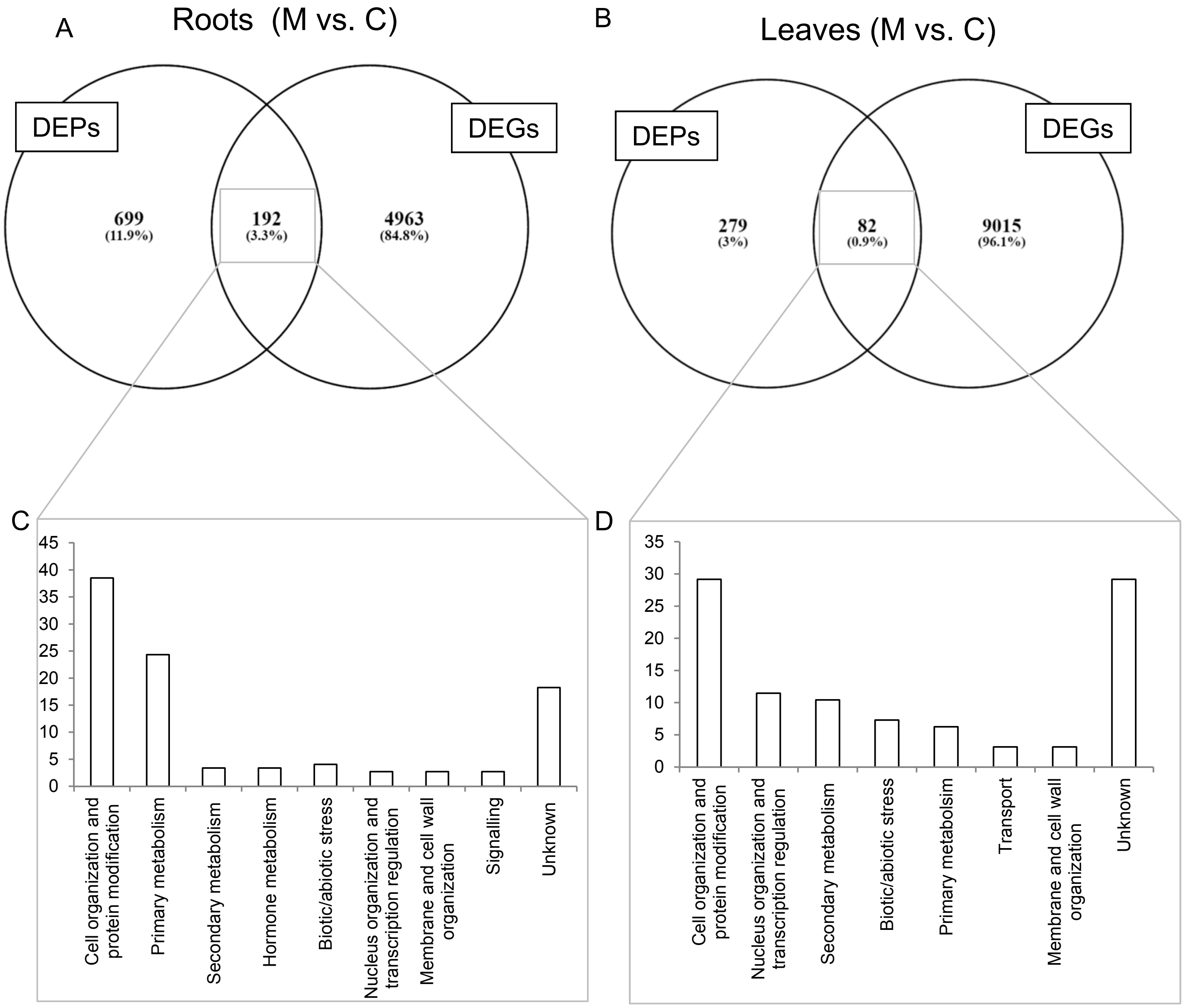

Fig. S2: Venn diagrams of control and AM fungal-modulated genes and proteins (DEGs and DEPs) in roots and leaves.

Omics approaches revealed how arbuscular mycorrhizal symbiosis enhances yield and resistance to leaf pathogen in wheat

Fiorilli Valentina, Vannini Candida, Ortolani Francesca, Garcia-Seco Daniel, Chiapello Marco, Novero Mara, Domingo Guido, Terzi Valeria, Morcia Caterina, Bagnaresi Paolo, Moulin Lionel, Bracale Marcella, Bonfante Paola.

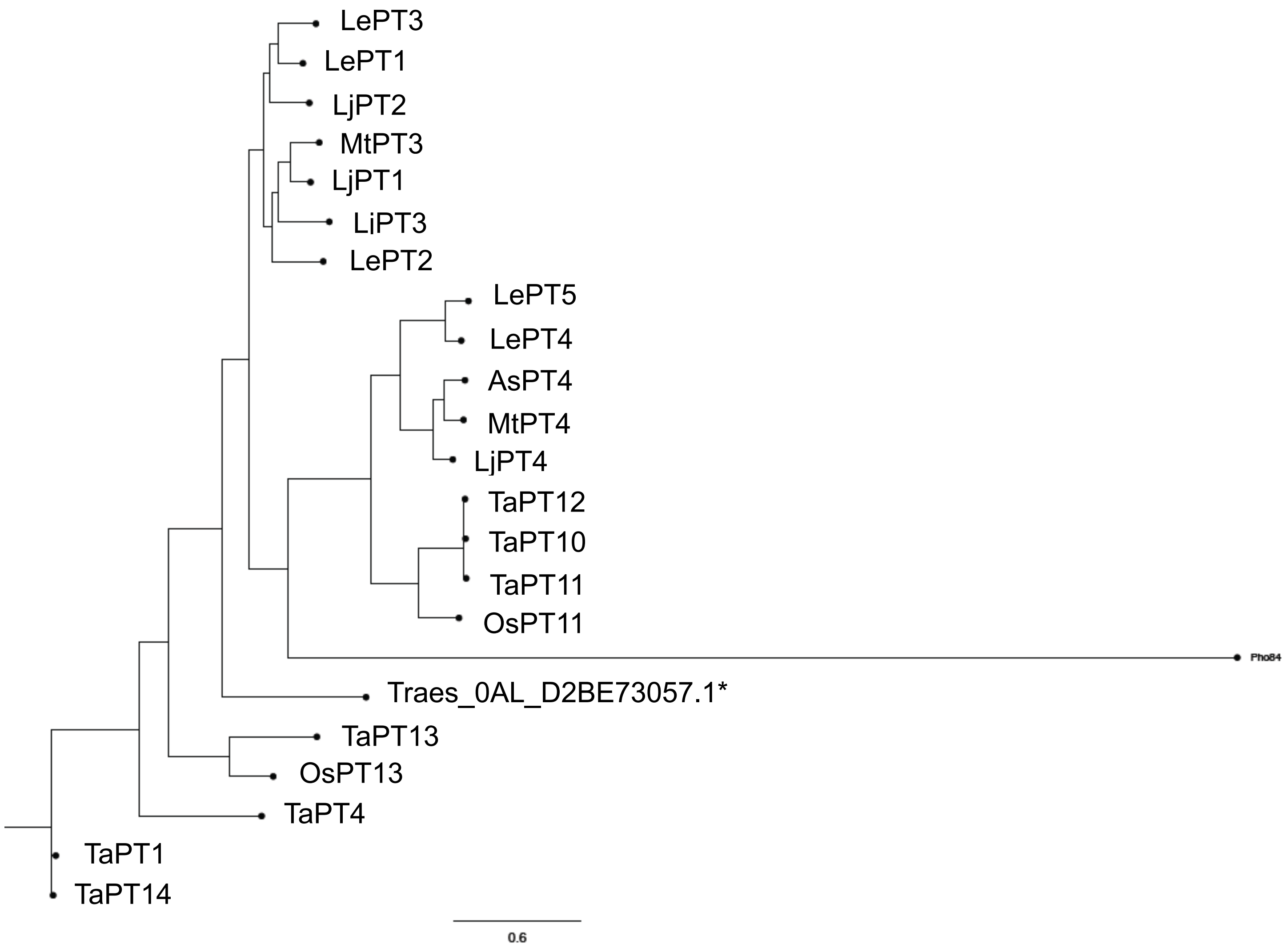

Fig. S3: The evolutionary history of the mycorrhiza-induced phosphate transporter (PT) genes.

Omics approaches revealed how arbuscular mycorrhizal symbiosis enhances yield and resistance to leaf pathogen in wheat

Fiorilli Valentina, Vannini Candida, Ortolani Francesca, Garcia-Seco Daniel, Chiapello Marco, Novero Mara, Domingo Guido, Terzi Valeria, Morcia Caterina, Bagnaresi Paolo, Moulin Lionel, Bracale Marcella, Bonfante Paola.

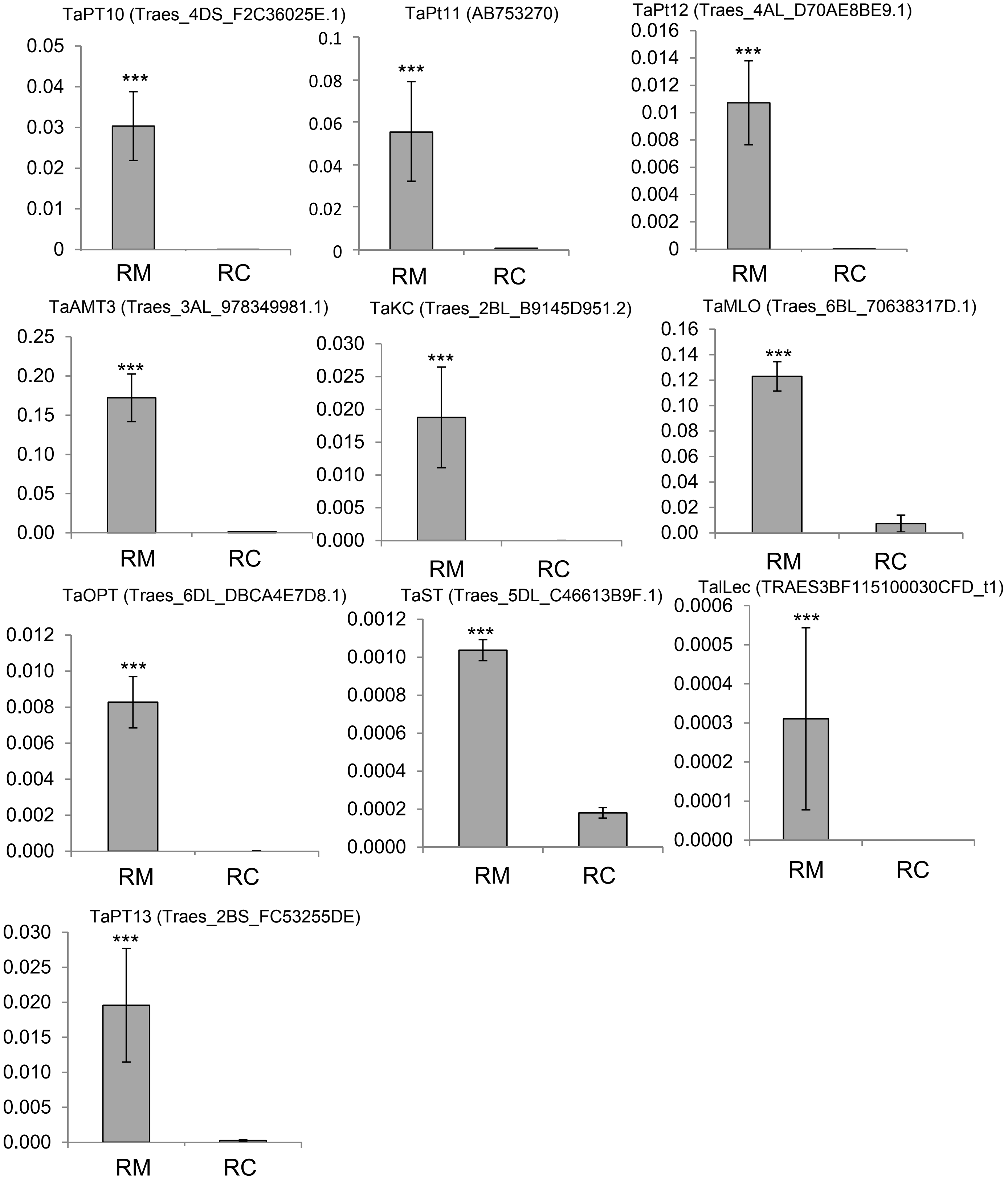

Fig. S4: Relative gene expression of ten differentially expressed genes.

Omics approaches revealed how arbuscular mycorrhizal symbiosis enhances yield and resistance to leaf pathogen in wheat

Fiorilli Valentina, Vannini Candida, Ortolani Francesca, Garcia-Seco Daniel, Chiapello Marco, Novero Mara, Domingo Guido, Terzi Valeria, Morcia Caterina, Bagnaresi Paolo, Moulin Lionel, Bracale Marcella, Bonfante Paola.

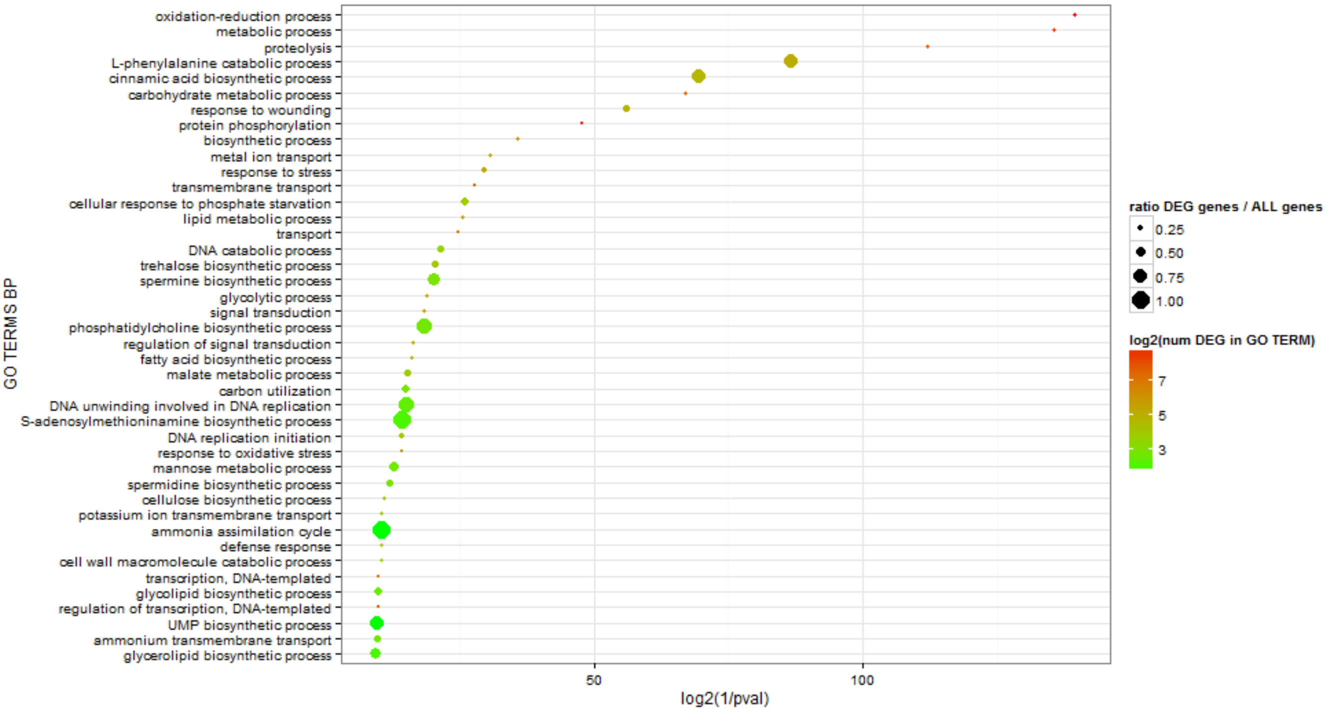

Fig. S5: Plots illustrating enriched GO terms specific for RM vs RC contrast.

Omics approaches revealed how arbuscular mycorrhizal symbiosis enhances yield and resistance to leaf pathogen in wheat

Fiorilli Valentina, Vannini Candida, Ortolani Francesca, Garcia-Seco Daniel, Chiapello Marco, Novero Mara, Domingo Guido, Terzi Valeria, Morcia Caterina, Bagnaresi Paolo, Moulin Lionel, Bracale Marcella, Bonfante Paola.

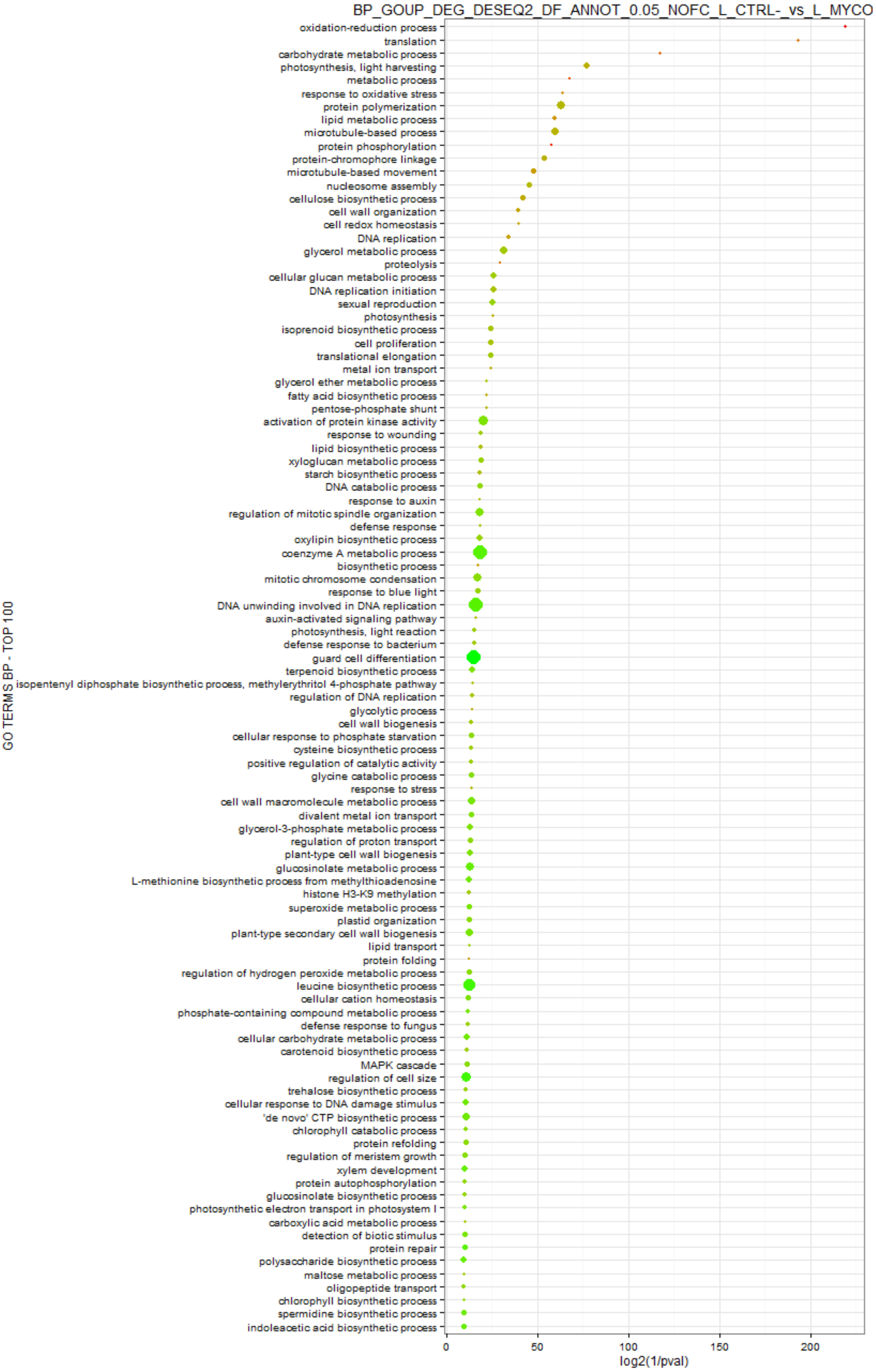

Fig. S6: Plots illustrating enriched GO terms specific for LM vs LC contrast.

Omics approaches revealed how arbuscular mycorrhizal symbiosis enhances yield and resistance to leaf pathogen in wheat

Fiorilli Valentina, Vannini Candida, Ortolani Francesca, Garcia-Seco Daniel, Chiapello Marco, Novero Mara, Domingo Guido, Terzi Valeria, Morcia Caterina, Bagnaresi Paolo, Moulin Lionel, Bracale Marcella, Bonfante Paola.

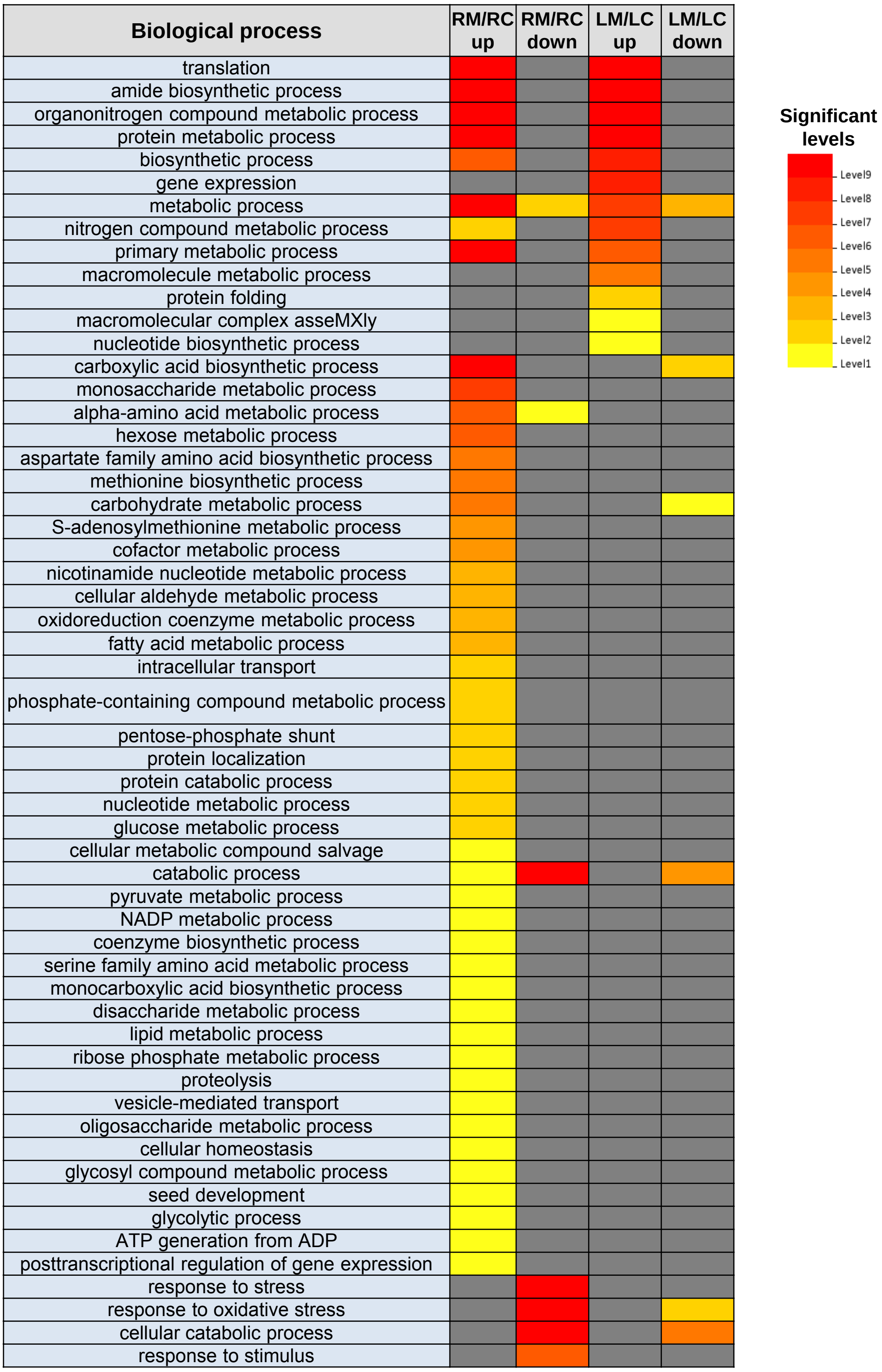

Fig.S7: Single enrichment analysis of protein GO terms specific for RM vs. RC and LM vs. LC contrasts.

Omics approaches revealed how arbuscular mycorrhizal symbiosis enhances yield and resistance to leaf pathogen in wheat

Fiorilli Valentina, Vannini Candida, Ortolani Francesca, Garcia-Seco Daniel, Chiapello Marco, Novero Mara, Domingo Guido, Terzi Valeria, Morcia Caterina, Bagnaresi Paolo, Moulin Lionel, Bracale Marcella, Bonfante Paola.

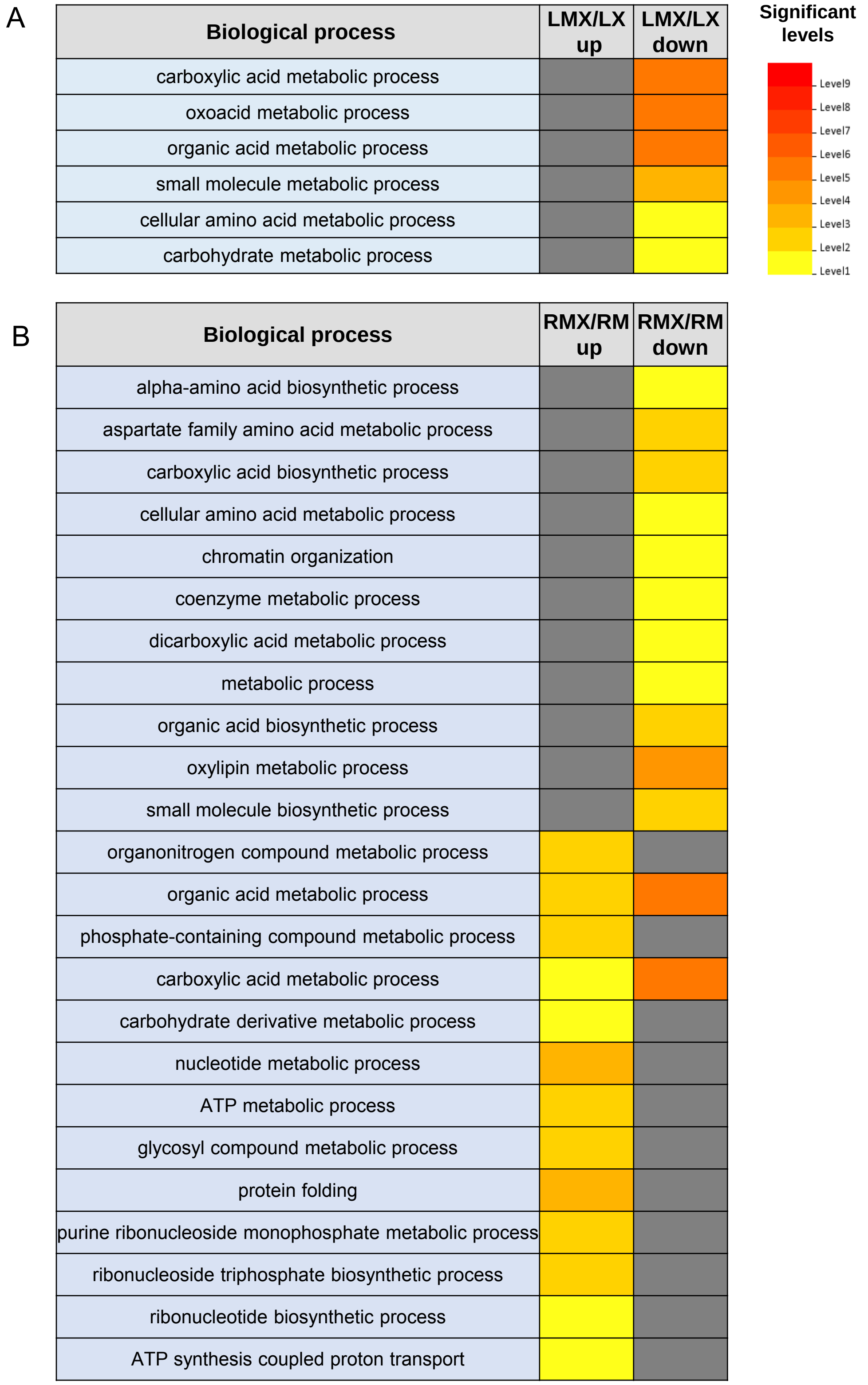

Fig. S8: Single enrichment analysis of protein GO terms specific for LMX vs. LX (A) and RMX vs. RM (B) contrasts.
